# miR‐15a‐5p and miR‐21‐5p contribute to chemoresistance in cytogenetically normal acute myeloid leukaemia by targeting PDCD4, ARL2 and BTG2

**DOI:** 10.1111/jcmm.16110

**Published:** 2020-12-03

**Authors:** Virginie Vandewalle, Ahmed Essaghir, Emeline Bollaert, Sandrine Lenglez, Carlos Graux, Hélène Schoemans, Pascale Saussoy, Lucienne Michaux, Peter J. M. Valk, Jean‐Baptiste Demoulin, Violaine Havelange

**Affiliations:** ^1^ Department of Hematology Cliniques Universitaires Saint‐Luc Brussels Belgium; ^2^ Experimental Medicine Unit de Duve Institute Université Catholique de Louvain Brussels Belgium; ^3^ Department of Hematology CHU UCL Namur (Godinne site) Yvoir Belgium; ^4^ Department of Hematology University Hospitals Leuven and KU Leuven Leuven Belgium; ^5^ Laboratory of Hematology Cliniques Universitaires Saint Luc Brussels Belgium; ^6^ Center for Human Genetics University Hospitals Leuven and KU Leuven Leuven Belgium; ^7^ Department of Hematology Erasmus University Medical Center Rotterdam the Netherlands

**Keywords:** acute myeloid leukaemia, apoptosis, chemoresistance, microRNAs, target genes

## Abstract

Cytarabine and daunorubicin are old drugs commonly used in the treatment of acute myeloid leukaemia (AML). Refractory or relapsed disease because of chemotherapy resistance is a major issue. microRNAs (miRNAs) were incriminated in resistance. This study aimed to identify miRNAs involved in chemoresistance in AML patients and to define their target genes. We focused on cytogenetically normal AML patients with wild‐type *NPM1* without *FLT3*‐ITD as the treatment of this subset of patients with intermediate‐risk cytogenetics is not well established. We analysed baseline AML samples by small RNA sequencing and compared the profile of chemoresistant to chemosensitive AML patients. Among the miRNAs significantly overexpressed in chemoresistant patients, we revealed *miR‐15a‐5p* and *miR‐21‐5p* as miRNAs with a major role in chemoresistance in AML. We showed that *miR‐15a‐5p* and *miR‐21‐5p* overexpression decreased apoptosis induced by cytarabine and/or daunorubicin. *PDCD4*, *ARL2* and *BTG2* genes were found to be targeted by *miR‐15a‐5p*, as well as *PDCD4* and *BTG2* by *miR‐21‐5p*. Inhibition experiments of the three target genes reproduced the functional effect of both miRNAs on chemosensitivity. Our study demonstrates that *miR‐15a‐5p* and *miR‐21‐5p* are overexpressed in a subgroup of chemoresistant AML patients. Both miRNAs induce chemoresistance by targeting three pro‐apoptotic genes *PDCD4*, *ARL2* and *BTG2*.

## 1 INTRODUCTION

Acute myeloid leukaemia (AML) is a heterogeneous group of clonal myeloid neoplasms with still a poor prognosis.^1^ For several decades, the standard of care for AML patients has been the classical “7 + 3” regimen combining 7 days of cytarabine and 3 days of an anthracycline.^2^, ^3^ During these years, the incremental improvements in AML patient overall survival that were observed were mainly because of better supportive care. Unfortunately, up to 30%‐40% of patients will develop a chemorefractory disease, defined as failure to achieve morphological complete response (CR) after one to two cycles of induction chemotherapy. This subgroup of AML patients faces a dismal prognosis with a median survival of <1 year.^4^, ^5^ To decipher underlying mechanisms of resistance to induction chemotherapy in AML, a better understanding of the heterogeneity of refractory patients at the cellular and molecular levels is critical. The development of novel therapies is very much needed to improve outcome of chemoresistant AML patients.

Acute myeloid leukaemia is the result of a clonal multistep evolutionary process starting with the acquisition of founder mutations in the haematopoietic stem cell followed by driver mutations ultimately leading to the development of AML.^2^ The cause of drug resistance can be intrinsic in patients who fail the initial chemotherapy because of the presence of multiples subclones or acquired after chemotherapy through the selection or acquisition of mutations in resistant subclones. Several pathways were altered in resistance including DNA damage and repair, cell cycling, cell death, drug targets, drug metabolism and drug trafficking.^6^, ^7^ Targeted therapies directed to the chemotherapy resistance mechanisms may improve the outcome for these patients.

microRNAs (miRNAs) constitute a class of short, evolutionary conserved, non‐coding RNAs with major regulatory functions. miRNAs can regulate up to 60% of protein‐coding genes. By binding to the 3′ untranslated region of target mRNAs, a single miRNA can negatively regulate the expression of a wide variety of target genes (up to 200 mRNAs). miRNA expression was found deregulated in human cancers and in particular in AML.^8^, ^9^ Each AML subtype exhibits a unique miRNA signature.^8^ Several studies showed that miRNA can function as tumour suppressor or onco‐miRNAs according to the roles of their target genes.^10^ miRNAs can affect crucial leukaemic processes such as proliferation, survival, differentiation, self‐renewal, epigenetic regulation, in vivo disease progression and chemotherapy resistance. Modifying miRNA expression is an exciting potential therapeutic approach in AML. Because miRNA can repress several targets involved in different pathways, miRNA‐based therapy would be an efficient tool against chemoresistance.

microRNAs were reported to regulate sensitivity and resistance to cytotoxic agents in AML cell lines.^7^, ^11^ Increased expression of some miRNAs resulted in chemosensitivity, whereas others were linked to chemoresistance. The mechanisms whereby miRNAs cause chemoresistance in AML patients are not well understood. Equally unclear are the molecular bases for differential expression of miRNAs in histologically and genetically similar AMLs. Understanding how miRNAs are differently expressed, their targets and effects on the AML clone might also help to better understand the leukemogenesis process.

In the current study, we assessed whether miRNAs play a role in chemoresistance in cytogenetically normal AML patients treated with standard induction chemotherapy. We analysed miRNA expression by high‐throughput small RNA sequencing in two cohorts of AML patients. Next, we performed functional tests on cell lines and identified target genes involved in chemoresistance. These results pave the way for therapeutic interventions to circumvent chemotherapy resistance.

## 2 MATERIALS AND METHODS

### 2.1 Patient samples

Frozen diagnostic bone marrow RNA samples were obtained from 47 adults who had a confirmed diagnosis of AML. Twenty‐seven of these patients were considered “chemosensitive” because they reached CR after receiving one cycle of induction chemotherapy. The twenty remaining patients were considered “chemoresistant”, defined as more than 5% blast cells in the bone marrow after induction chemotherapy. Cytogenetic analyses were performed at diagnosis. *FLT3*‐ITD and *NPM1* mutation analysis was performed by PCR amplification and sequencing. Patients with a cytogenetically normal AML (CN‐AML) and wild‐type *NPM1* without *FLT3‐ITD* were included in the study (Table S1). *TP53*, *ASXL1* and *RUNX1* mutational status was obtained for 37 patients by next‐generation sequencing (Illumina TruSight Myeloid Panel) as previously described.^12^

Patient clinical characteristics are shown in Table S1.

Blasts and mononuclear cells at diagnosis were purified by Ficoll‐Hypaque (Nygaard) density gradient centrifugation and cryopreserved. RNA was isolated with either RNA‐Bee or RLT following the protocols of the manufacturer (Bio‐Connect BV). Frozen diagnostic bone marrow RNA samples were analysed by small RNA sequencing.

### 2.2 Small RNA sequencing

Multiplexed patient libraries (TruSeq small RNA) were prepared and sequenced within the same flow cell (HiSeq 2000 PE 2 × 50 bp sequencing). Samples were run in the Genomic Core Lab Facility in UZ Leuven.

See supplemental methods (Appendix S1) for details.

### 2.3 Cell lines

The human AML cell lines K562 and OCI‐AML3 (purchased from DSMZ) were cultured in RPMI 1640 supplemented with 10% foetal bovine serum and in Alpha‐MEM with 20% foetal bovine serum, respectively, and with 50 U/mL penicillin and 50 mg/mL streptomycin (Gibco). Cells were treated with cytarabine (Ara‐C; Sigma #C1768), daunorubicin (DNR; Sigma #W4013) or vehicle (water or DMSO). K562 cells were treated with daunorubicin 0.5 µmol/L and/or cytarabine 5 µmol/L for 24 hours. OCI‐AML3 cells were treated with daunorubicin 0.1 µmol/L and/or cytarabine 1 µmol/L for 24 hours as these cells are more sensitive to chemotherapy.

### 2.4 Generation of stable miRNA‐expressing cell lines using lentiviral infection

The lentiviral‐expressing vector of endogenous miR‐15a and miR‐21 human pre‐miRNAs was purchased from Systems Biosciences (# PMIRH15aPA‐1 and PMIRH21PA‐1). See supplemental methods (Appendix S1) for details.

The efficiency of the infection was checked by measuring the green fluorescent protein (GFP) expression by flow cytometry and was around 88% and 72% in K562 and OCI‐AML3, respectively, as shown in Figure S1A. miRNA mature expression was measured by qRT‐PCR (Figure S1B). An empty vector was used as control.

### 2.5 Transient transfection

The synthetic *miR*Vana™ hsa‐miR‐15a‐5p mimic (#MC10235) and *miR*Vana™ hsa‐miR‐21‐5p mimic (#MC10206) were purchased from Life Technologies. A total of 5 million of K562 cells were nucleoporated using Amaxa^®^ Nucleofector^®^ Technology (Lonza) with 100 µL of solution V (#VCA‐1003; Program T‐016) with 500 pmol of precursor oligonucleotide. The transfection efficiency of K562 cells using this method is close to 90%. *miR‐15a‐5p* and *miR‐21‐5p* expression levels after transfection without and with daunorubicin and cytarabine treatment are shown in Figure S2. Scrambled oligonucleotides were used as control.

### 2.6 Functional assays

Annexin V (V450)—BV421 stain (BD Biosciences Pharmingen; #563973)—was performed 24h after the treatment and measured using FACSVerse.

### 2.7 Microarray studies on cell lines

Microarrays were performed according to the Affymetrix^®^ WT PLUS standard protocol following the manufacturer’s instructions.

See supplemental methods (Appendix S1) for details.

### 2.8 Quantitative reverse transcription polymerase chain reaction

For the quantification of miRNA levels after lentiviral infections, reverse transcription was carried out using the QuantiMir Reverse Transcription Kit (System Biosciences, #RA420A‐1) and real‐time PCR was performed using the designed forward primer, the universal reverse primer, the SYBR Green qPCR Master Mix and the Quantimir cDNA according to the manufacturer’s instructions. Normalization was completed with *U6*. Comparative real‐time polymerase chain reaction (PCR) was performed in triplicate. Quantitative data were calculated using a regression curve from serial dilutions of a standard cDNA.

For the quantification of miRNA levels in other experiments, 50 ng of total RNA was used for reverse transcription, using TaqMan MicroRNA Reverse Transcription Kit (Life Technologies) and real‐time PCR was performed using the TaqMan MicroRNA Assay Kit (Life Technologies; miR‐15a‐5p #000397 and miR‐21‐5p #000389) according to the manufacturer's instructions. Normalization was completed with small nucleolar *RNU44* (Life Technologies*;* *#*001094). Relative expression was calculated using the comparative cross‐threshold (Ct) method.

For the quantification of mRNA levels, 1 µg of total RNA was used for reverse transcription, using MMLV (Moloney murine leukaemia virus) reverse transcriptase enzyme (Invitrogen). We used TaqMan Gene Expression assays (Life Technologies; PDCD4 #Hs00377253, ARL2 #Hs00155873, BTG2 3Hs0019888). *GAPDH* served as normalization control (Life Technologies; Hs02758991). Comparative real‐time PCR was performed in triplicate. Relative expression was calculated using the comparative cross‐threshold (Ct) method.

### 2.9 Western blotting

Total protein extracts from K562 cells transfected with synthetic miRNAs and scrambled oligonucleotides were extracted using lysis buffer (25 mm Tris‐HCl, pH 7.4, 150 mmol/L NaCl, 6 mmol/L EDTA, 10% glycerol and 1% Triton X‐100) containing protease inhibitors (1 mmol/L Pefabloc^®^, 1.7 μg/mL aprotinin and 1 mmol/L orthovanadate de sodium). Protein expression was analysed by Western blotting using PDCD4 (Santa Cruz, sc#130545) and ARL2 (Abcam, ab#109742) antibodies as previously described.^13^

### 2.10 Luciferase reporter experiments

The 3′UTR segments containing the target sites for *miR‐15a‐5p* and *miR‐21‐5p* were amplified by PCR from cDNA and inserted into pGL3 control vector (Promega), using the *Xba*1 site.

See supplemental methods (Appendix S1) for details.

### 2.11 Gene expression profiles of patient samples

Expression levels of the three target genes—*PDCD4* (1557166 at, 202730 s at, 202731 at, 212593 s at, 212594 at), *ARL2* (202564 x at) and *BTG2* (201235 s at, 201236 s at)—were analysed in 43 on a total of 47 patient samples by using Affymetrix U133A GeneChips as previously described.^14^

### 2.12 PCA and correlation studies

A principal component analysis (PCA) using gene and miRNA expression data for the selected miRNAs (*miR‐15a‐5p* and *miR‐21‐5p*) and probesets of *PDCD4*, *ARL2* and *BTG2* genes was performed in R program using the mixOmics package.^15^ Chemoresistant patient numbers 59, 61, 62 and 63 were removed from the PCA because we did not have enough RNA left to perform gene expression profiles. To study the correlation between genes and miRNAs that were the basis of this PCA, correlation plot was generated from the PCA. Normal regression analysis was performed between the clinical data and the patient loadings on the three first PCs. Each clinical phenotype (bone marrow blast percentage, white blood cell count, platelet count) was evaluated individually against the patient loading on the three PCs.

### 2.13 siRNA transfections

A total of 5 million of K562 cells were nucleoporated using Amaxa^®^ Nucleofector^®^ Technology (Lonza) with 100 µL of solution V (#VCA‐1003; Program T‐016) with 50 nmol/L anti‐PDCD4 (# L‐004438‐00‐0020) and with 50 nmol/L anti‐ARL2 (# L‐011585‐00‐0020) and with 50 nmol/L anti‐BTG2 (# L‐012308‐00‐005) compared with a control (non‐targeting siRNA #1 #D‐001810‐01‐20) (Dharmacon, SMARTpool siRNAs or control). The efficiency of the siRNAs was evaluated by Western blotting for PDCD4 and ARL2 and by qRT‐PCR for the three target genes at 24 hours (Figure S3).

## 3 RESULTS

### 3.1 *miR‐15a‐5p* and *miR‐21‐5p* are overexpressed in chemoresistant AML patients

To identify miRNAs that are involved in chemoresistance, we first analysed miRNA expression in bone marrow samples from 47 AML patients at diagnosis, before treatment with cytarabine and anthracycline, using Illumina small RNA sequencing. We selected samples from patients younger than 65 years with cytogenetically normal AML (CN‐AML) and wild‐type *NPM1* without *FLT3*‐ITD. This subgroup of AML patients was included in the intermediate‐risk category by the 2010 ELN genetic risk stratification.^16^ The consolidation therapy of the intermediate‐risk genetics category is not well established. The three options proposed by the ELN are intensive chemotherapy, high‐dose therapy followed by autologous or allogeneic haematopoietic stem cell transplantation.^3^, ^16^

We compared pre‐treatment miRNA expression in chemosensitive (n = 27) and chemoresistant (n = 20) patients. Relevant miRNAs were sorted based on three criteria (their relative linear fold change, area under the ROC curve and frequency of random forest feature selection) as computed by the procedures explained in material and methods. Table S3 showed the list of miRNAs differentially expressed between both groups of patients with a highly significant *P*‐value (≤10E‐08) and a frequency of selection of 100%.

We selected the four miRNAs of this list with a linear fold change above 7 (Table 1).

**Table 1:** Most significant miRNAs differentially expressed between chemosensitive and chemoresistant patient groups (linear fold change > 7)

Mature miRNAs (ID)	Linear fold change resistant/sensitive	*P*‐value	Linear fold change based on geometric mean resistant/sensitive	AUC (ROC curve)	Random Forest feature selection frequency (%)	Validation by qRT‐PCR with a significant difference
hsa‐miR‐21‐5p (MIMAT0000076)	18.65	2.50E‐014	4.26	0.833	100	Yes
hsa‐miR‐340‐5p (MIMAT0004692)	11.79	2.59E‐011	3.6	0.846	100	No
hsa‐miR‐181c‐5p (MIMAT0000258)	7.62	7.99E‐008	3.26	0.817	100	No
hsa‐miR‐15a‐5p (MIMAT0000068)	7.21	2.15E‐011	3.22	0.886	100	Yes

Note

The selection frequency represents the number of times a miRNA was ranked among the top 30 selected features discriminating the resistant/sensitive phenotype by the leave‐one‐out cross‐validated random forest model, using the absolute T statistics ranking.

**Figure 1:**
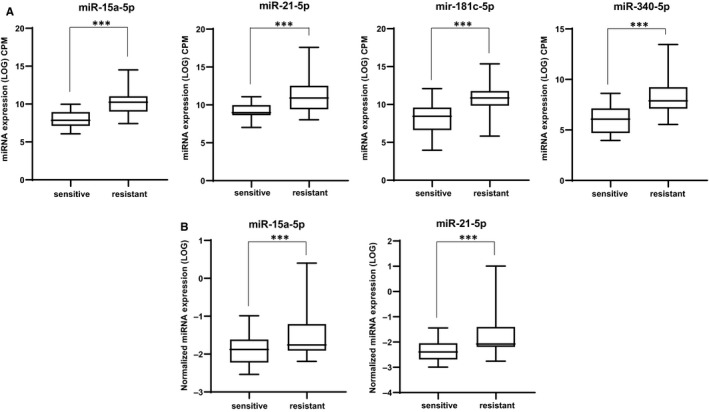
*miR‐21‐5p*, *miR‐340‐5p*, *miR‐181c‐5p* and *miR‐15a‐5p* are overexpressed in chemotherapy‐resistant AML patients. A, Expression of *miR‐21‐5p*, *miR‐340‐5p*, *miR‐181c‐5p* and *miR‐15a‐5p* was analysed by small RNA sequencing in AML patients, with normal karyotype, sensitive (n = 27) or resistant (n = 20) to the standard chemotherapy combining cytarabine and daunorubicin. The box plot is based on geometric mean (log_10_ CPM). *P* values were obtained using a *t* test. *** Indicates a significant difference *P* *≤* .001. B, Expression of *miR‐15a‐5p* and *miR‐21‐5p* was analysed by qRT‐PCR in AML patients, with normal karyotype, sensitive (n = 27) or resistant (n = 20) to standard chemotherapy treatment combining cytarabine and daunorubicin. The box plot is based on geometric mean (log_10_). *P* values were obtained using a *t* test.*** Indicates a significant difference *P* *≤* .001

Figure 1A shows the distribution of the expression of *miR‐21‐5p*, *miR‐340‐5p*, *miR‐181c‐5p* and *miR‐15a‐5p*. The distribution confirmed the difference between both groups of patients. We validated the significant differential expression of two of these miRNAs—*miR‐15a‐5p* and *miR‐21‐5p*—by qRT‐PCR in patient samples (Figure 1B). We did not find a significant differential expression of *miR‐340‐5p* and *miR‐181c‐5p* between the two groups of patients by qRT‐PCR (data not shown).

The more recent ELN2017 genetic risk stratification specified that this subgroup of AML patients with wild‐type *NPM1* and no *FLT3*‐ITD (or with *FLT3*‐ITD low) was classified in the intermediate‐risk category if they did not have any adverse‐risk genetic lesion. We collected the mutational status for *RUNX1*, *ASXL1* and *TP53* in eighty percent of our patients and excluded the patients with *RUNX1* or *ASXL1* mutation. *TP53* mutation was not found in our patients. The overexpression of *miR‐21‐5p* and *miR‐15a‐5p* in chemoresistant AML patients regarding chemosensitive patients was confirmed in our smaller cohort of patients classified in the intermediate‐risk category following the ELN2017 genetic risk stratification (Figure S4).

We compared patient clinical characteristics at diagnosis (bone marrow blast percentage, white blood cell count, platelet count) in chemoresistant and chemosensitive AML patients (Table S1). The regression analysis did not show any significant correlation between the clinical characteristics and *miR‐21a‐5p* or *miR‐15a‐5p* expression level or the chemoresistance (data not shown).

### 3.2 *miR‐15a‐5p* and *miR‐21‐5p* reduce apoptosis induced by cytarabine and daunorubicin

The expression *of* *miR‐15a‐5p* and *miR‐21‐5p* was first measured by qRT‐PCR in K562 and OCI‐AML3 cell lines. *miR‐21‐5p* was expressed at a higher level than miR‐*15a‐5p* in both cell lines. The treatment of K562 or OCI‐AML3 with daunorubicin or with cytarabine did not significantly change *miR‐15a‐5p* or *miR‐21‐5p* expression (Figure S5).

**Figure 2:**
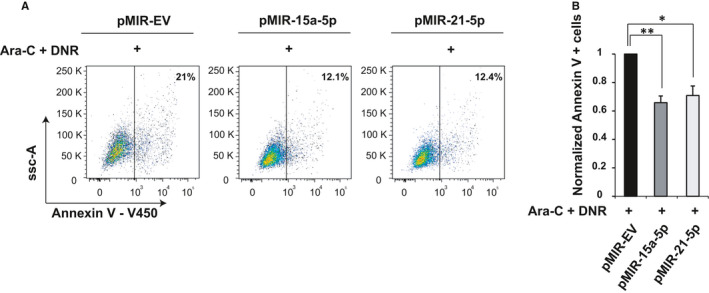
*miR‐15a‐5p* and *miR‐21‐5p* reduce apoptosis induced by the combination of cytarabine and daunorubicin. K562 cells were infected with lenti‐miR virus to overexpress *miR‐15a‐5p*, *miR‐21‐5p* or EV. Annexin V assay was performed after 24 h of treatment with cytarabine (Ara‐C) and daunorubicin (DNR). The results are presented as percentage of apoptotic cells. One representative experiment is shown in (A) and the average of th independent experiments ± SD in (B). *P* values were obtained using a *t* test. * Indicates a significant difference *P* *≤* .05, and ** indicates a significant difference *P* *≤* .01

To evaluate the impact of *miR‐15a‐5p* and *miR‐21‐5p* on chemosensitivity, we stably overexpressed both miRNAs in the cell lines (K562 and OCI‐AML3) using a lentiviral construct containing the pre‐miR‐15a‐5p or pre‐miR‐21‐5p. The empty lentivirus was used as control. The efficiency of the infection was measured by analysing the GFP expression by flow cytometry and miRNA mature expression by qRT‐PCR (Figure S1). Infected cells were treated with cytarabine and/or daunorubicin for 24 hours. As shown in Figure 2, the overexpression of *miR‐15a‐5p* or *miR‐21‐5p* significantly decreased apoptosis induced by the combination of cytarabine and daunorubicin in K562. The overexpression of *miR‐15a‐5p* or *miR‐21‐5p* also decreased apoptosis induced by cytarabine alone or by daunorubicin alone (Figure S6). Like in K562, in OCI‐AML3, the overexpression of either miRNAs decreased apoptosis induced by cytarabine and daunorubicin alone or combined (Figure S7).

As a note, note that the overexpression of *miR‐15a‐5p* or *miR‐21‐5p* did not lead to a significant reduction in apoptosis in the absence of chemotherapy in both cell lines (Figure S8).

Remarkably, enforced expression of *miR‐15a‐5p* and *miR‐21a‐5p* by lentiviral infection caused only a 5‐ to 7‐fold increase in expression of miRNAs, which was comparable to the difference in expression that we observed between chemosensitive and chemoresistant patients. Our observations suggest that a 5‐ to 7‐fold change in *miR‐15a‐5p* and *miR‐21‐5p* expression was sufficient to play an important role in apoptosis and chemoresistance.

### 3.3 miRNA target genes involved in chemoresistance

To investigate potential *miR‐15a‐5p* and *miR‐21‐5p* target genes involved in chemoresistance, we overexpressed each miRNA in K562 cell line and simultaneously treated the cells with daunorubicin using a concentration of 1 µmol/L. Synthetic miRNA oligonucleotides and controls (scrambled oligonucleotides) were transfected in K562 using nucleoporation. The transfection efficiency was controlled 24 hours after transfection by measuring miRNA expression using qRT‐PCR (data not shown). Gene expression profiles were determined 24 hours after transfection and daunorubicin treatment by using Affymetrix microarrays. The lists of down‐regulated mRNAs were crossed with target genes identified by bioinformatic prediction programs (PicTar and TargetScan). We found 30 genes that were down‐regulated by *miR‐21‐5p* and were predicted as target genes by PicTar and TargetScan (Table S4). After overexpression of *miR‐15a‐5p*, we found 141 down‐regulated genes that were predicted as target genes by both programmes (Table S4). The most down‐regulated genes were *BTG2* and *PDCD4* after overexpression of both miRNAs, and *ARL2* after overexpression of *miR‐15a‐5p* only (Table 2).

**Table 2:** List of most down‐regulated mRNAs after *miR‐15a‐5p* or *miR‐21‐5p* overexpression in K562 cells

Probe Set ID	mir21 vs scramble log‐ratio	mir21 vs scramble log‐ratio	Gene symbol	Gene description
TC01001685.hg.1	−0.206044	−0.707511	BTG2	BTG family, member 2
TC10000808.hg.1	−0.193792	−0.420942	PDCD4	Programmed cell death 4 (neoplastic transformation inhibitor)

Probe Set ID	mir15 vs scramble log‐ratio	mir15 vs scramble log‐ratio	Gene symbol	Gene description
TC11003457.hg.1	−1.104408	−1.205423	ARL2	ADP ribosylation factor‐like 2
TC01001685.hg.1	−0.028212	−0.888098	BTG2	BTG family, member 2
TC10000808.hg.1	−0.268	−0.843751	PDCD4	Programmed cell death 4 (neoplastic transformation inhibitor)

To validate these three potential *miR‐15a‐5p* and *miR‐21‐5p* target genes, we measured their expression by quantitative RT‐PCR and by western blotting after ectopic expression of *miR‐15a‐5p* and *miR‐21‐5p*. Increased level of both miRNAs upon transfection was confirmed by qRT‐PCR (Figure S2A).

**Figure 3:**
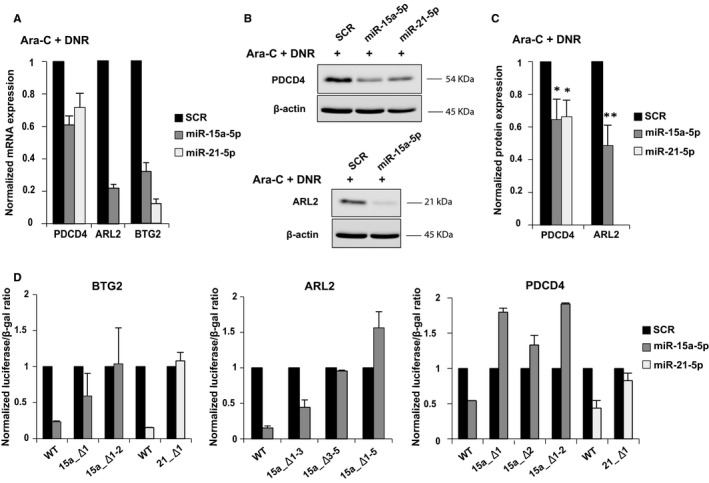
*miR‐15a‐5p* and *miR‐21‐5p* regulate the expression of ARL2, PDCD4 and BTG2. A, Quantitative RT‐PCR of *PDCD4*, *ARL2* and *BTG2* in K562 after 24 h of transfection with synthetic miR‐15a‐5p, miR‐21‐5p or SCR treated with cytarabine and daunorubicin. The results are shown as average mRNA expression after normalization with *GAPDH* and 2ΔC_t_ calculations. B, Western blotting of PDCD4 and ARL2 protein expression in K562 cells treated with cytarabine and daunorubicin after 24 h of transfection with synthetic *miR‐15a‐5p*, *miR‐21‐5p* or SCR. The protein loading control was performed using β‐actin. C, Bands were quantified by densitometry using Image J software. Data represent the average of three independent experiments ± SD. *P* values were obtained using a *t* test. * Indicates a significant difference *P* *≤* .05, and ** indicates a significant difference *P* *≤* .01. D, Luciferase activity in HEK293 cells cotransfected with synthetic miR‐15a‐5p, miR‐21‐5p or SCR and luciferase reporter constructs containing wild‐type (WT) or mutated (miRNA_Δ) *PDCD4*, *ARL2* and *BTG2* 3′UTR. Luciferase activities were determined at 24 h and were normalized using β‐galactosidase activity. The mutant plasmids were generated by deleting the *miR‐15a‐5p* or *miR‐21‐5p* binding site(s)

*miR‐15a‐5p* and *miR‐21‐5p* overexpression significantly reduced *ARL2*, *PDCD4* and *BTG2* mRNA expression in K562 cells treated with cytarabine and daunorubicin (Figure 3A). The overexpression of *miR‐15a‐5p* or *miR‐21‐5p* in K562 cells treated with cytarabine and daunorubicin resulted in down‐regulation of PDCD4 at the protein level with respect to the scrambled control (Figure 3B,C). ARL2 expression was also decreased after overexpression of *miR‐15a‐5p* only (Figure 3B,C). Noticeably, we could not detect BTG2 expression by western blotting by processing with the available commercial antibodies. The same results were obtained in K562 cell lines treated with cytarabine alone or daunorubicin alone versus vehicle (Figure S9).

We cloned the 3′UTR of *ARL2*, *PDCD4* and *BTG2* containing the seed sequences that were predicted to interact with *miR‐15a‐5p* and *miR‐21‐5p* into a luciferase reporter vector pGL3 control (Figure S10). We cotransfected the luciferase reporter with the synthetic miRNA of interest into K562 cell line. There are two predicted interaction sites for *miR‐15a‐5p* in the *PDCD4* 3′UTR, five in the *ARL2* 3′UTR and two in the *BTG2* 3′UTR (Figure S10). The *miR‐21‐5p* has one predicted interaction site in the *PDCD4* 3′UTR and one in the *BTG2* 3′UTR (Figure S10). A significant reduction in the luciferase/βgal ratio was observed for *ARL2*, *PDCD4* and *BTG2* constructs transfected with synthetic miRNA compared with the control (Figure 3D). This effect was abrogated when we cotransfected mutated 3′UTR luciferase reporter vectors and the synthetic miRNA. The mutations were created by the deletion of 4‐6 bases in the seed sequence of the miRNAs (Figure S10). These results confirmed the direct regulation of the three target genes by *miR‐15a‐5p* and/or *miR‐21‐5p*.

### 3.4 Target gene validation in patient samples

**Figure 4:**
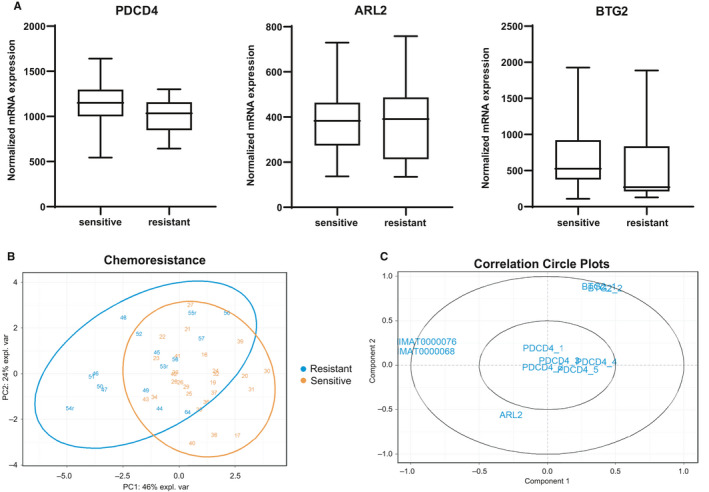
Target gene validation in patient samples. A, Expression of *PDCD4*, *ARL2* *and* *BTG2* was analysed by gene expression profiles (Affymetrix) in AML patients, with normal karyotype, sensitive (n = 27) or resistant (n = 16) to standard chemotherapy treatment combining cytarabine and daunorubicin. The box plot is based on microarray normalized mRNA expression. B, PCA based on selected gene and miRNA expression data were performed in R program using the mixOmics package. The PCA plot of PC2 to PC1 is shown in (B). The correlation plot of the miRNAs and genes used to perform the PCA is shown in (C)

To validate the three target genes in our patient cohort, we analysed the data from the gene expression profiles performed on patient samples. Figure 4A shows the down‐regulation of the three target genes in chemoresistant AML patients compared with chemosensitive. The same results were observed in our smaller cohort of patients after the exclusion of the patients with *ASXL1* and *RUNX1* mutation following the ELN2017 risk stratification (data not shown).

We performed a PCA based on selected gene and miRNA expression data. The first three principal components explained eighty‐four per cent of the variance. Figure 4B shows the PCA plot of PC2 to PC1 (the two principal components). Individuals such as “54r”, “50”, “47”, “46” and “48” are chemoresistant and clearly separated from the rest. Individuals “20”, “31”, “30”, “40”, “36” and “17” are chemosensitive and clearly separated from the rest. A separation of the two phenotypes (chemoresistant: the left side of the plot versus chemosensitive: the right side of the plot) can be observed. However, the separation between the phenotypes is still hard to achieve for some patients (the middle of the plot). The PC3 versus PC1 plot captured the separation of the same chemoresistant individuals that were clearly separated from the chemosensitive individuals in the PC2 versus PC1 plot (Figure S11).

Figure 4C shows the correlation plot of the selected miRNAs and genes. The two miRNAs were clustered together, and the probesets were clustered per their corresponding genes. The plot shows a negative correlation based on the axis of PC1 between the two miRNAs and the *BTG2* gene. A negative correlation between the two miRNAs and the *ARL2* gene is also demonstrated based on the PC2 axis. For the *PDCD4* gene, some probesets show more negative correlation with the two miRNAs according to the PC1 axis. In summary, based on the combination of the two first principal components we could distinguish a negative correlation between the expression of the two miRNAs and the three target genes.

### 3.5 *miR‐15a‐5p* and *miR‐21‐5p* induce chemoresistance by targeting *ARL2*, *PDCD4* and *BTG2*

**Figure 5:**
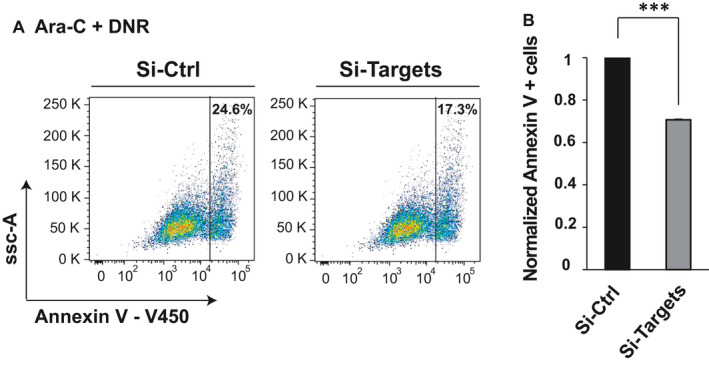
*miR‐15a‐5p* and *miR‐21‐5p* induce chemoresistance by targeting ARL2, PDCD4 and BTG2. K562 cell lines were transfected with a combination of three siRNAs against the three target genes (siPDCD4, siARL2, siBTG2) at a concentration of 50 nmol/L during 48 h. K562 cells were treated with cytarabine (Ara‐C) and daunorubicin (DNR) the last 24 h. The results are presented as percentage of apoptotic cells. One representative experiment is shown in (A) and the average of three independent experiments ± SD in (B). *P* values were obtained using *t* test. ***Indicates a significant difference *P* *≤* .001

To confirm the direct roles of the three target genes in chemoresistance, we silenced *ARL2*, *PDCD4* and *BTG2* in K562 cells. K562 cells were transfected with a combination of three siRNAs against the three target genes (siARL2, siPDCD4 and siBTG2) at a concentration of 50 nmol/L each. We used a reduced concentration as the common 100 nmol/L concentration was too toxic for the cell line. The efficiency of the siRNAs was evaluated by western blotting for PDCD4 and ARL2 and by qRT‐PCR for the three target genes at 24 hours (Figure S3). After 24 hours of transfection, K562 cells were treated with cytarabine and daunorubicin. Chemosensitivity was assessed by apoptosis by an Annexin V test. The percentage of Annexin‐positive cells significantly decreased after the combined inhibition of the three target genes (Figure 5). This was not observed after the inhibition of only two of the three target genes such as *PDCD4* and *ARL2* (data not shown). The same effect on chemosensitivity was observed after treatment of the cells with cytarabine or daunorubicin alone (Figure S12). In conclusion, inhibiting the three target genes reproduced the functional effects of both miRNAs.

## 4 DISCUSSION

Acute myeloid leukaemia is only cured in 35%‐40% of young adults. The main problem remains resistance to the old commonly used chemotherapy combining cytarabine and an anthracycline, that is daunorubicin. This paper provides new evidence of the important roles of miRNAs in chemoresistance in cytogenetically normal AML patients mediated by target genes implicated in early apoptosis.

In the present study, we demonstrated that *miR‐15a‐5p* and *miR‐21‐5p* are significantly overexpressed in a cohort of chemoresistant compared with chemosensitive cytogenetically normal AML patients with wild‐type *NPM1* without *FLT3*‐ITD. Our gain‐of‐function experiments in AML cell lines showed that *miR‐15a‐5p* or *miR‐21‐5p* overexpression decreases apoptosis after daunorubicin and/or cytarabine treatment to almost reach the level of untreated cells. We found three interesting genes with a pro‐apoptotic function. We confirmed that *PDCD4*, *BTG2* and *ARL2* are responsible for *miR‐15a‐5p* and *miR‐21‐5p* modulating effect on chemosensitivity of leukaemic cells to cytarabine and daunorubicin. The effect on chemosensitivity was stronger when the cells were treated with the combination of cytarabine and daunorubicin such as the treatment of the patients than with each drug alone. The most differentially down‐regulated genes after *miR‐15a‐5p* or *miR‐21‐5p* overexpression in our AML cell lines treated with daunorubicin are *PDCD4*, *BTG2* and *ARL2*. It seems that the expression of both miRNAs was positively correlated in our patient series supporting a synergistic role on the same target genes and on chemoresistance. No common mechanism of deregulation has been described for both miRNAs. Moreover, a negative correlation between the expression of both miRNAs and the three target genes was confirmed in patient samples. Our data suggest that several miRNAs can modulate chemosensitivity of myeloid leukaemia cells to cytarabine and/or daunorubicin by targeting the same genes.

Based on PCA performed on miRNA and gene expression, some patients could be phenotypically separated in chemoresistant or chemosensitive. Although we observed a tendency for phenotype separation, other patients could not be segregated as their expression profile for the selected genes and miRNAs was intermediate. Mechanisms affecting their expression need to be further explored. We cannot exclude additional mechanisms of chemoresistance in this group of unsegregated patients.

These three target genes have been previously linked to chemoresistance. *PDCD4* (programmed cell death 4) is a tumour suppressor gene that not only inhibits proliferation, migration and invasion but also promotes apoptosis in tumours.^17^ Zhang et al. showed that *PDCD4* overexpression activate BAX (member of BCL2 protein family) that induces apoptosis in hepatoma cells.^18^ Knockdown of *PDCD4* was shown to promote resistance to chemotherapy in different types of cancer cells, and *PDCD4* was a functional target for *miR‐21*–induced chemoresistance in tongue squamous carcinoma cells.^19^ *ARL2* (ADP ribosylation factor‐like 2) is a small GTP protein of the RAS superfamily whose increased expression was correlated with reduced aggressiveness of breast tumour cells.^20^ Breast cancer cells expressing low level of *ARL2* were not sensitive to contact inhibition, had a stronger clonogenic potential and an enhanced tumour growth.^20^ Decreased *ARL2* expression was associated with resistance to cytotoxic agents in breast cancer cell lines.^21^ In glioma cells, Wang et al. showed that *ARL2* is an important suppressor of proliferation.^22^ *BTG2* (B cell translocation gene 2) and *BTG1* (B cell translocation gene 1) belong to the B cell translocation gene family of anti‐proliferation proteins and regulate apoptosis, cell cycle progression and differentiation. The expression of *BTG1* was induced in CR AML patients and was decreased in refractory AML patients.^23^ Interestingly, Tong et al reported that *miR‐21* could protect cardiomyocytes against doxorubicin‐induced apoptosis by targeting *BTG2*, confirming our observations.^24^

*miR‐21* has been shown to drive chemoresistance by targeting *PDCD4*, *PTEN* or other genes in several solid tumours, such as renal carcinoma, hepatocellular carcinoma, colon carcinoma and gastric cancer cells.^25^, ^26^, ^27^ *miR‐21* was also found consistently up‐regulated in AML blasts compared with normal CD34+ cells.^28^ No study had specifically investigated the role of *miR‐21* in chemoresistance in AML patients, but Bai et al found in 2011 that *miR‐21* overexpression in AML cell lines induced daunorubicin resistance in K562 by targeting *PTEN*.^29^ Li et al^30^ reported that *miR‐21* inhibition sensitizes AML cell lines to cytarabine by inducing apoptosis. These effects seemed to be partially because of the up‐regulation of *PDCD4*.^30^ *miR‐15a* was initially found down‐regulated in a subgroup of chronic lymphocytic leukaemia patients.^31^ Little is known about the role of *miR‐15a* in chemoresistance. In AML patients, high expression of *miR‐15a* has been shown to predict shorter survival and worse response to chemotherapy.^32^

Finally, there is an urgent need to develop new therapeutic approaches in AML. miRNAs can modulate multiple signalling pathways and regulatory networks in AML.^33^ Slight changes in miRNA expression could be responsible for significant changes in treatment response. Modifying miRNA expression may target different pathways in AML blasts and avoid the development of chemoresistance. *miR‐15a‐5p* and *miR‐21‐5p* could be used as future targets for therapy. Synthetic miRNAs are now involved in phase 1 and phase 2 clinical trials. For example, MRG‐106 is an LNA antagomir that targets miR‐155 and is now tested in the treatment of selected types of lymphoma or leukaemia.^34^ Our results suggested a promising further therapeutic option that will combine anti‐miR‐15a‐5p and/or anti‐miR‐21‐5p with standard induction chemotherapy to improve remission rate and decrease relapse rate mainly in chemoresistant AML patients.

## CONFLICT OF INTEREST

The authors confirm that there are no conflicts of interest.

## AUTHOR CONTRIBUTIONS

**Virginie Vandewalle:** Conceptualization (equal); Data curation (equal); Investigation (lead); Methodology (equal); Validation (equal). **Ahmed Essaghir:** Conceptualization (equal); Data curation (equal); Formal analysis (equal); Methodology (equal); Software (lead). **Emeline Bollaert:** Conceptualization (equal); Data curation (equal); Investigation (equal); Methodology (equal); Validation (equal). **Sandrine Lenglez:** Conceptualization (equal); Data curation (equal); Investigation (equal); Validation (equal). **Carlos Graux:** Resources (equal); Writing‐review & editing (equal). **Hélène Schoemans:** Resources (equal); Writing‐review & editing (equal). **Pascale Saussoy:** Resources (equal); Writing‐review & editing (equal). **Lucienne Michaux:** Resources (equal); Writing‐review & editing (equal). **Peter Valk:** Resources (equal); Writing‐original draft (equal). **Jean‐Baptiste Demoulin:** Supervision (equal); Writing‐original draft (equal). **Violaine Havelange:** Conceptualization (lead); Data curation (lead); Formal analysis (lead); Funding acquisition (lead); Investigation (lead); Methodology (lead); Project administration (lead); Resources (lead); Supervision (lead); Writing‐original draft (supporting).

## Supporting information

Supplementary Material

## Data Availability

Small RNA‐sequencing data (fastq files) are available in the ArrayExpress database (http://www.ebi.ac.uk/arrayexpress ^35^) under accession number E‐MTAB‐8775. Microarray data are available in the ArrayExpress database (http://www.ebi.ac.uk/arrayexpress ^35^) under accession number E‐MTAB‐8776.
