# Supplementary material for: miR‐15a‐5p and miR‐21‐5p contribute to chemoresistance in cytogenetically normal acute myeloid leukaemia by targeting PDCD4, ARL2 and BTG2
Source: J Cell Mol Med. 2020 Dec 3;25(1):575–85. doi: 10.1111/jcmm.16110 (PMC7810923; doi:10.1111/jcmm.16110)
Supplement: Supplementary file 1 — Supplementary Material [file JCMM-25-575-s001.docx]

**Supplemental methods, figures, and tables.**

1. **Supplemental material and methods.**
2. **Supplemental figures and tables.**

**1. Supplemental material and methods**

**Appendix S1**

**Patient samples**: The study was approved by our hospital ethical committee.

On the forty-seven patients, forty-five patients were included in an HOVON clinical

trial and signed the HOVON informed consent. For the last two patients, the board

waived the need for an informed consent as the study was retrospective based on

the use of samples of residual human body material.

**Small RNA-sequencing**: The raw data consisted of per lane fastq file for each patient. The fastq files were merged by AML patient. Fastq files contained the 51 sequenced bases of each read and their corresponding quality scores, which reflect the sequencing error probability. The quality control of each fastq file was examined using the fastqc software ^1^. We mainly focused on the distribution of quality scores by position within all reads. All samples had median scores per position above 30, reflecting a base call error probability below 0.001. We then used the cutadapt tool ^2^ to trim the 5' and 3' adapters, tolerating 10% mismatched error rate within a matched adapter sequence and discarding all remaining reads having a length below 8 bases after the adapter cut. The remaining reads were aligned to the GRCh37 release version of the human reference genome, downloaded from NCBI genome database. The alignment was performed using the BWA program ^3^, tolerating one mismatch within the seed region, for which the length parameter was set to 8. The resulting alignment files were then converted to bam format before controlling their quality using the fastqc program. Using R programming functions, we parsed the bam files for reads that were uniquely mapped to the mature miRNAs, as annotated within miRbase. We then build a matrix of read counts per miRNA within each patient. This matrix contained raw counts data for mature miRNAs that were expressed in at least one patient. The Rsamtools and GenomicRanges R packages were used for these tasks ^4,5^.

To prioritize miRNAs that could be differentially expressed in the chemoresistant patients relative to the chemosensitive group, we used the three follow-up procedures: (i) the miRNA count matrix was fed to a standard edgeR workflow ^6^, which normalizes and fits a negative binomial generalized linear model to the data. Based on this, the significantly differentially expressed mature miRNAs between the chemoresistant and the chemosensitive groups were determined. These miRNAs were then ranked according to their geometric mean expression log-ratio and the p-value; (ii) we tested the capability of these individual miRNA expression to distinguish between the two groups of patients, based on the area under the ROC curve (AUC) analysis as implemented in the genefilter R package ^7^, keeping only mature miRNAs having AUCs above 0.7; (iii) Using a MLInterfaces R package ^8^, we applied to these miRNAs a machine learning procedure consisting of a random forest model within a “leave one out” cross validation and using the top 30 absolute t-statistic rank as a feature selection. This procedure gave an overall error rate ~ 22%. MiRNAs were then ranked according to their selection frequencies. Geometric means, ROC curve and the machine learning procedure were based on data normalized with the voom method as implemented in the limma R package ^9^.

**Generation of stable miRNA expressing cell lines using lentiviral infection:**

Lentiviral particles were produced by HEK-293T cells as described ^10^. Cells were seeded in T-75 flasks. After one day, plasmid DNA (18 µg) was co-transfected with the packaging plasmids pCMV-dr8.2 dvpr (10 µg), pCMV-VSV-G (6 µg) and pRSV-Rev (6 µg). The DNA was mixed with 750 µl of BBS buffer (50 mM N,N-bis-(2-hydroxyethyl)-2-aminoethane-sulfonic acid, pH 7, 280 mM NaCl, 1.5 mM Na2HPO4) and 75 µl CaCl_2_ 2.5 M. The solution was incubated for 20 min at room temperature. Chloroquine (25 mM, Sigma #C6628) was added to HEK-293T cells for 20 min at 37 °C. Precipitates (1.5 ml) were added to each plate. Four hours after transfection, cells were washed and incubated in medium during 48 h. Supernatants were concentrated with Centricon technology (Merckmillipore; Centricon® Plus-70 Centrifugal filter #UFC703008) and used to infect the cells.

**Microarray studies on cell lines**: 100 ng of total RNA was used as a starting material. The GeneChip WT PLUS Reagent Kit (Affymetrix ®) was used for ss-cDNA preparation, fragmentation, and labeling. Human Transcriptome Arrays 2.0 (HTA 2.0) chips were used for hybridization. The hybridization, wash and scan were done according to the Affymetrix kits and procedures specific to the HTA 2.0 chips. After the scan, the quality controls of the hybridization were checked using the Gene Expression Console software (Affymetrix ®). The RMA-Sketch procedure was used for data normalization. Fold change was determined for each comparison based on these normalized values. We used the DAVID web tool ^11^ for functional annotation and pathway analysis of the selected gene lists.

**Luciferase reporter experiments**: We generated mutant inserts with deletions of 5 bp from the site of perfect complementarity using the QuickChange XL-II kit (Agilent) according to the manufacturer’s protocol. Mutagenic primers were synthesized by Eurogentec and are listed in supplemental Table S2. Wild-type and mutant inserts were verified by sequencing. K562 cells were cotransfected using nucleoporation with 0.5 µg of firefly luciferase reporter vector and 0.5 µg of pEF1-β-galactosidase (Invitrogen) as internal control and synthetic miRNA or the scrambled oligonucleotides. After 24h, cells were lysed by passive lysis buffer (Promega) and the luciferase activity was measured using a GloMax® instrument (Turner Biosystems). The β-galactosidase activity was assessed as described^12^. The data are represented as the average ratio between the luciferase and the β-galactosidase activities and normalized to the empty vector condition.

**2.Supplemental figures and tables.**

| **Characteristics** | **Chemosensitive patients (n=27)** | **Chemoresistant patients**  **(n=20)** |
| --- | --- | --- |
| **Age**  Median | 41 | 46 |
| **Sex** (%)  Female  Male | 10 (37%)  17 (63%) | 12 (60%)  8 (40%) |
| **Bone marrow blasts** (%)  Median | 51 | 75 |
| **Baseline WBC count** (x10^9^/L)  Median | 7.6 | 32 |
| **Baseline platelet count** (x10^9^/L)  Median | 46 | 55 |
| **Prior chemotherapy**  No  Yes | 25  2 | 20  0 |
| **Prior MDS**  No  Yes | 26  1 | 20  0 |

**Supplemental Table S1. Patient characteristics.**


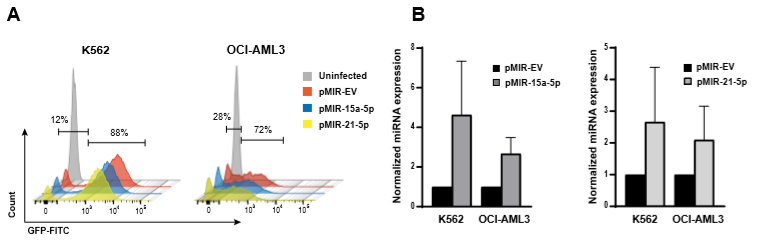


**Supplemental Figure S1. Lentivirus infection efficiency and *miR-15a-5p* and *miR-21-5p* expression in K562 and OCI-AML3 cell lines.**

K562 and OCI-AML-3 cells were infected with lentimiR virus to overexpress *miR-15a-5p*, *miR-21-5p* or the empty vector. Green fluorescein protein (GFP) expression of one representative infection measured by flow cytometry is represented in (**A**). Quantitative RT-PCR (qRT-PCR) of *miR-15a-5p* and *miR-21-5p* expression was measured after lentivirus infection. The results are shown as miRNA expression levels after normalization with *U6* and a regression curve from serial dilutions of a standard cDNA in (**B**). Data represent the average of 3 independent experiments ± SD.

**
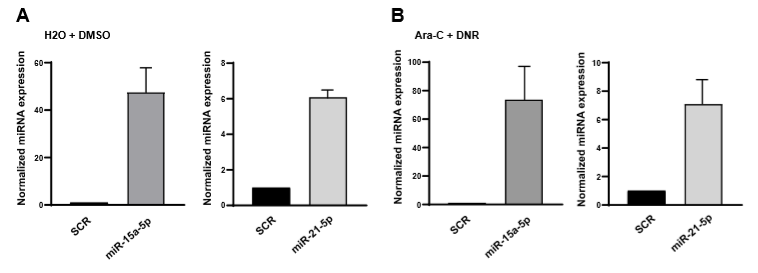
**

**Supplemental Figure S2. Expression levels of *miR-15a-5p* and *miR-21-5p* in K562 cell lines after transient transfection.**

Quantitative RT-PCR (qRT-PCR) of *miR-15a-5p* and *miR-21-5p* expression after transfection with synthetic miR-15a-5p or miR-21-5p or scrambled oligonucleotides (SCR) in K562 cell line without treatment (**A**) or after 24 hours of treatment with daunorubicin 0,5 µM and cytarabine 5 µM (**B**). Three independent experiments were performed. The results of one representative experiment are shown in (**A**) and (**B**) and are represented as miRNA expression levels after normalization with *RNU44* and 2ΔCt calculations. Bars represent SD.

|  | **Sens (5’-3’)** | **Antisense (5’-3’)** |
| --- | --- | --- |
| **Primers for 3’UTR wild-type construct** | | |
| **ARL2** | **TCTAGACAACCTTCACCAAACACTACC** | **TCTAGACAACTGAGTGAAGGATGAGGCC** |
| **BTG2** | **TCTAGAGGCCTGTAGATGTTGCTTTC** | **TCTAGATAACGTGACATTCTTCCAT** |
| **PDCD4** | **TCTAGAGCAAGGAGGGACAGAAAAGTAAC** | **TCTAGAAGACCAATCAGTATGTTCCCTGG** |
| **Primers for 3’UTR del construct** | | |
| **ARL2_15a_ Δ1+2** | **CCTCCACCCCAGCCTACTGCTGCCCG** | **CGGGCAGCAGTAGGCTGGGGTGGAGG** |
| **ARL2_15a_Δ3** | **TACTGCTGCCCGCTTCTGTGGCCACC** | **GGTGGCCACAGAAGCGGGCAGCAGTA** |
| **ARL2_15a_Δ4+5** | **GGCGGGGAGGAGACCGAGGCTGTG** | **CACAGCCTCGGTCTCCTCCCCGCC** |
| **BTG2_15a_Δ1** | **CAAAACCTTTGCTTTGCTAGTTTTGTGTGTATGTGTGGCAAA** | **TTTGCCACACATACACACAAAACTAGCAAACGAAAGGTTTTG** |
| **BTG2_15a_Δ2** | **GGAACCACATGAAAGTCTTGATTGCCATGATCCC** | **GGGATCATGGCAATCAAGACTTTCATGTGGTTCC** |
| **BTG2_21-Δ1** | **GTAAAATAAATAGTAGTAGTATGTTTGTATTCTGACAGAAAAGACAA** | **TTAGTAACCTTTGACTTTTCTGTCAGAATACAAACATACTACTACTATTTATTTTAC** |
| **PDCD4_15a_Δ1** | **TATTTAGGGGGTAAAGTTAAGAAAACCCCATGTTGGCTG** | **CAGCCAACATGGGGTTTTCTTAACTTTACCCCCTAAATA** |
| **PDCD4_15a_Δ2** | **CTAAAACCCCATGTTGGCTTGTTGAGATACTGTGC** | **GCACAGTATCTCAACAAGCCAACATGGGGTTTTAG** |
| **PDCD4_21_Δ1** | **AGTAACCTCTTCTTAAGTGGAATATTCTAATACCTTTTGTAAGTGCCA** | **TGGCACTTACAAAAGGTATTAGAATATTCCACTTAAGAAGAGGTTACT** |

**Supplemental Table S2.** Primers used to clone the wild type 3′ UTR of *ARL2, BTG2 and PDCD4* in the PGL-3 luciferase reporter vector are shown in the upper part of the table. Primers used to clone the 3′ UTR of *ARL2, BTG2 and PDCD4* with the miR-15a-5p or miR-21-5p interaction sites deleted in the PGL-3 luciferase reporter vector are shown in the lower part of the table.

**
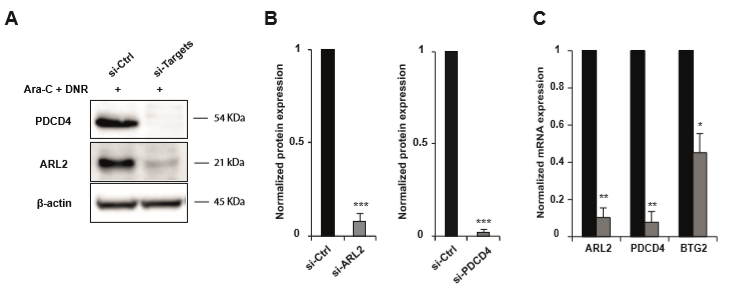
**

**Supplemental Figure S3.** ***ARL2, PDCD4* and *BTG2* silencing by siRNA transfection in K562 cell line.** K562 cell line were transfected with a combination of three siRNAs against the three target genes (siPDCD4, siARL2, siBTG2) at a concentration of 50 nM for 48 hours. K562 were treated with cytarabine (Ara-C) and daunorubicin (DNR) the last 24 hours. Protein expression is showed in (**A**). Bands were quantified by densitometry using Image J software in (**B**). Data represent the average of 3 independent experiments ± SD. *P* values were obtained using a *t* test. *** Indicates a significant difference *p ≤ 0.001*. mRNA expression is showed in (**C**). The results are shown after normalization with GAPDH and 2ΔCt calculations. Bars represent SD. *P* values were obtained using *t* test. * Indicates a significant difference *p ≤ 0.05 and* ** indicates a significant difference *p ≤ 0.01.*

| **MiRNA ID** | **MiRNA Name** | **linear fold change resistant/**  **sensitive** | **p-value** | **linear fold change geometric mean resistant/**  **sensitive** | **AUC**  **(ROC curve)** | **Selection frequency** |
| --- | --- | --- | --- | --- | --- | --- |
| MIMAT0000076 | hsa-miR-21-5p | 18,65 | 2,50E-014 | 4,26 | 0.833 | 100% |
| MIMAT0004692 | hsa-miR-340-5p | 11,79 | 2,59E-011 | 3,60 | 0.846 | 100% |
| MIMAT0000258 | hsa-miR-181c-5p | 7,61 | 7,99E-008 | 3,26 | 0.817 | 100% |
| MIMAT0000068 | hsa-miR-15a-5p | 7,21 | 2,15E-011 | 3,22 | 0.886 | 100% |
| MIMAT0000456 | hsa-miR-186-5p | 5,70 | 3,46E-009 | 3,10 | 0.795 | 100% |
| MIMAT0000680 | hsa-miR-106b-5p | 4,85 | 1,19E-009 | 3,08 | 0.875 | 100% |
| MIMAT0003339 | hsa-miR-421 | 4,45 | 4,43E-008 | 3,08 | 0.808 | 100% |
| MIMAT0000085 | hsa-miR-28-5p | 3,90 | 2,86E-008 | 3,07 | 0.894 | 100% |
| MIMAT0000081 | hsa-miR-25-3p | 3,52 | 9,58E-008 | 2,99 | 0.848 | 100% |

**Supplemental Table S3**. List of miRNAs differentially expressed between both groups of patients with a highly significant p-value (≤ 10E-08) and a frequency of selection of 100%.

**
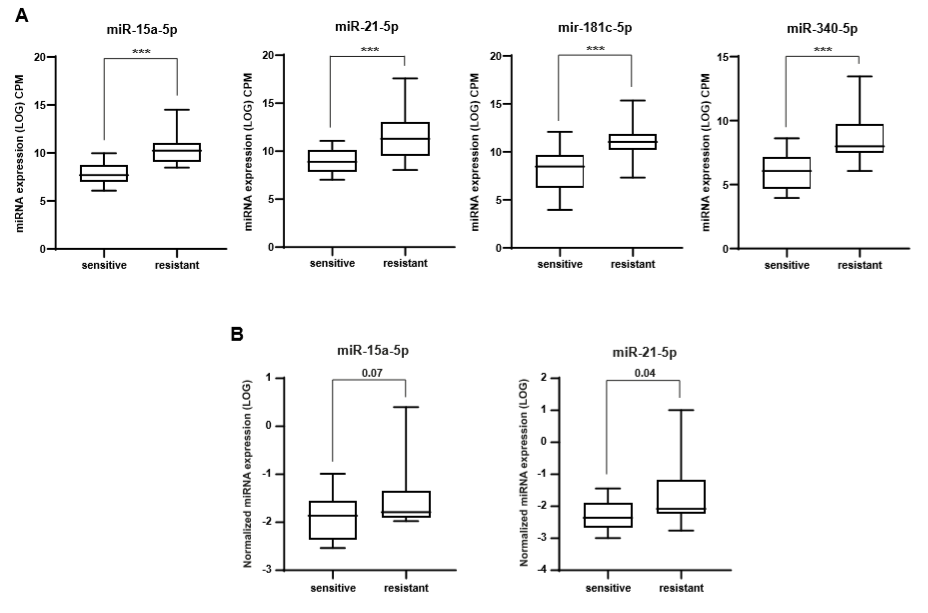
**

**Supplemental Figure S4. *miR-21-5p,* *miR-340-5p, miR-181c-5p* and *miR-15a-5p* are overexpressed in chemotherapy resistant AML patients classified in the intermediate risk category following the ELN2017 genetic risk stratification.**

(**A**) Expression of *miR-21-5p*, *miR-340-5p*, *miR-181c-5p* and *miR-15a-5p* was analyzed by small RNA-sequencing in AML sensitive (n=18) or resistant (n=8) to the standard chemotherapy combining cytarabine and daunorubicin. ELN2017 information’s were available for 23 chemosensitive and 14 chemoresistant patients. Patients must have a normal karyotype, wild-type *NPM1* without *FLT3*-ITD or with *FLT3*-ITD^low^ and no adverse-risk genetic lesions. Patients with *ASXL1* and/or *RUNX1* mutations were removed from the selection. The boxplot is based on geometric mean (Log10 CPM). *** Indicates a significant difference *p ≤ 0.001*.

(**B**) Expression of *miR-15a-5p* and *miR-21-5p* was analyzed by qRT-PCR in AML patients, with normal karyotype, sensitive (n=18) or resistant (n=8) to standard chemotherapy treatment combining cytarabine and daunorubicin. *P* values were obtained using *Wilcoxon test*.

**
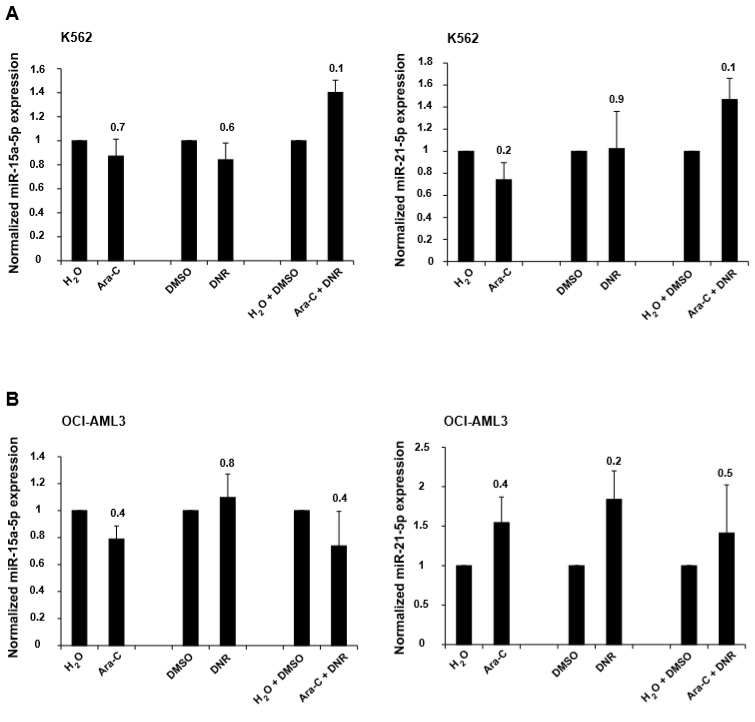
**

**Supplemental Figure S5. Cytarabine and/or daunorubicin treatment does not significantly change *miR-15a-5p* and *miR-21-5p* expression in K562 and OCI-AML3 cell lines.**

Quantitative RT-PCR (qRT-PCR) of *miR-15a-5p* and *miR-21-5p* expression was measured in K562 (**A**) and OCI-AML3 (**B**) cell lines. K562 cells were treated with daunorubicin 0.5 µM and/or cytarabine 5 µM for 24 hours. OCI-AML3 cells were treated with daunorubicin 0.1 µM and/or cytarabine 1 µM for 24 hours*.* The results are shown as miRNA expression levels after normalization with *RNU44* and 2ΔCt calculations. Bars represent SD. *P* values were obtained using a *t* test and are indicated on the graphs.


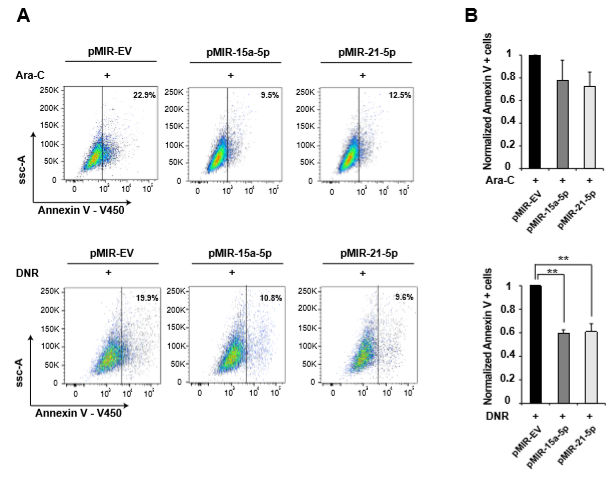


**Supplemental Figure S6**. ***miR-15a-5p* and *miR-21-5p* reduce apoptosis induced by cytarabine or by daunorubicin treatment.**

K562 cells were infected with lentimiR virus to overexpress *miR-15a-5p*, *miR-21-5p* or EV. Annexin V assay was performed after 24 hours of treatment with cytarabine (Ara-C) or daunorubicin (DNR). The results are presented as percentage of apoptotic cells. One representative experiment is showed in **(A)** and the average of 3 independent experiments ± SD in (**B)**. *P* values were obtained using *t* test. ** Indicates a significant difference *p ≤ 0.01*.

**
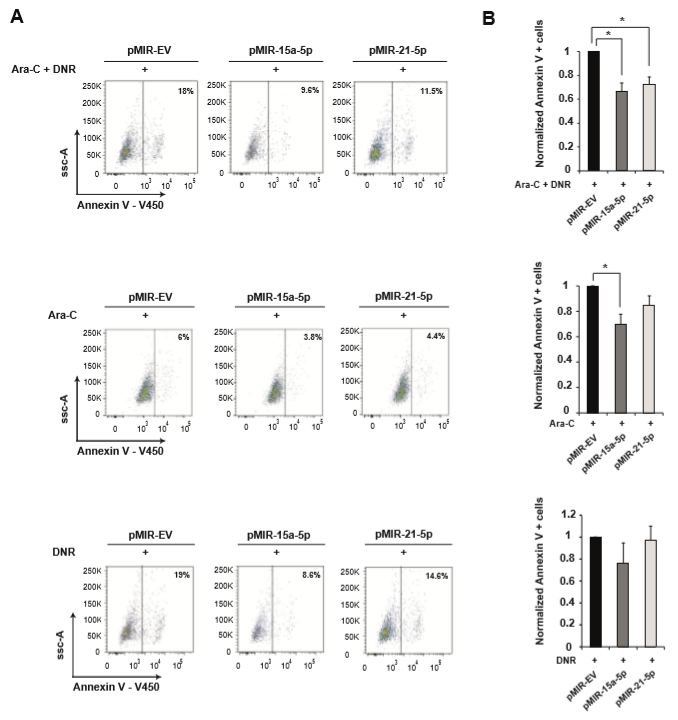
**

**Supplemental Figure S7**. ***miR-15a-5p* and *miR-21-5p* reduce apoptosis induced by cytarabine and/or daunorubicin treatment in OCI-AML3 cell line.** OCI-AML3 cells were infected with lentimiR virus to overexpress *miR-15a-5p,* *miR-21-5p* or EV. Annexin V assay was performed after 24 hours of treatment with cytarabine (AraC) or/and daunorubicin (DNR). The results are presented as percentage of apoptotic cells. One representative experiment is shown in **(A)** and the average of 3 independent experiments ± SD in (**B)**. *P* values were obtained using *t* test. * Indicates a significant difference *p ≤ 0.05*.


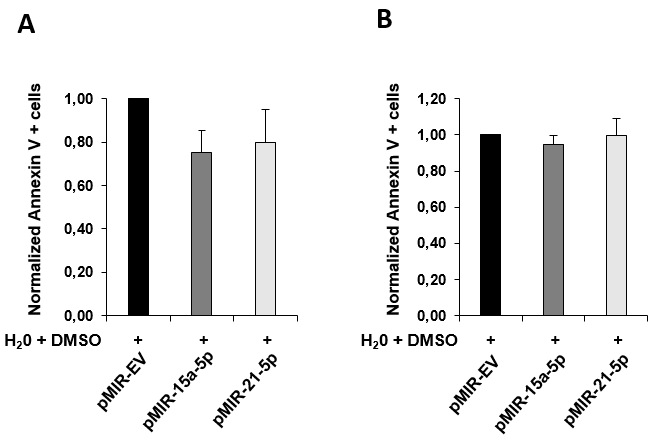


**Supplemental Figure S8. *miR-15a-5p* and *miR-21-5p* do not significantly change apoptosis without any chemotherapy**. K562 and OCI-AML3 cells were infected with lentimiR virus to overexpress *miR-15a-5p*, *miR-21-5p* or EV. Annexin V assay was performed after 24 hours of treatment with vehicle (water and DMSO). The results are presented as percentage of apoptotic cells. The average of three independent experiments ± SD is shown in K562 in **(A)** and in OCI-AML3 in **(B)**.

| Probe Set ID | **mir21 vs scramble log-ratio** | **mir21 vs scramble log-ratio** | Gene Symbol | Gene Description |
| --- | --- | --- | --- | --- |
| TC01001685.hg.1 | **-0,206044** | **-0,707511** | **BTG2** | **BTG family, member 2** |
| TC10000808.hg.1 | **-0,193792** | **-0,420942** | **PDCD4** | **programmed cell death 4 (neoplastic transformation inhibitor)** |
| TC0X000909.hg.1 | **-0,2199** | **-0,184123** | EIF1AX | eukaryotic translation initiation factor 1A, X-linked |
| TC08000538.hg.1 | **-0,174711** | **-0,165786** | WWP1 | WW domaincontaining E3 ubiquitinprotein ligase 1 |
| TC03001374.hg.1 | **-0,165223** | **-0,131846** | CDC25A | cell division cycle 25A |
| TC17001531.hg.1 | **-0,018793** | **-0,120853** | STAT3 | signal transducer and activator of transcription 3 (acute-phase response factor) |
| TC0X000222.hg.1 | **-0,063126** | **-0,116294** | RP2 | retinitispigmentosa 2 (X-linkedrecessive) |
| TC03000969.hg.1 | **-0,087364** | **-0,115976** | SOX2 | SRY (sex determining region Y)-box 2 |
| TC17001360.hg.1 | **-0,131935** | **-0,090443** | CCL1 | chemokine (C-C motif) ligand 1 |
| TC09001038.hg.1 | **-0,065451** | **-0,085315** | CNTFR | ciliaryneurotrophic factor receptor |
| TC13000566.hg.1 | **-0,170874** | **-0,075655** | SPG20 | spasticparaplegia 20 (Troyer syndrome) |
| TC11001915.hg.1 | **-0,015117** | **-0,066226** | EHD1 | EH-domaincontaining 1 |
| TC08002610.hg.1 | **-0,089744** | **-0,064143** | SOX7 | SRY (sex determining region Y)-box 7 |
| TC15002009.hg.1 | **-0,050286** | **-0,06036** | PCSK6 | proproteinconvertasesubtilisin/kexin type 6 |
| TC04000466.hg.1 | **-0,112454** | **-0,057701** | ARHGAP24 | Rho GTPaseactivatingprotein 24 |
| TC15000609.hg.1 | **-0,138734** | **-0,048501** | RAB11A | RAB11A, member RAS oncogene family |
| TC12001307.hg.1 | **-0,011404** | **-0,048468** | SOX5 | SRY (sex determining region Y)-box 5 |
| TC10001526.hg.1 | **-0,245551** | **-0,038649** | CPEB3 | cytoplasmicpolyadenylationelement binding protein 3 |
| TC12000176.hg.1 | **-0,151482** | **-0,031155** | CREBL2 | cAMP responsive element binding protein-like 2 |
| TC09000201.hg.1 | **-0,036367** | **-0,027834** | RECK | reversion-inducing-cysteine-rich protein with kazal motifs |
| TC22000391.hg.1 | **-0,000485** | **-0,026609** | PPARA | peroxisome proliferator-activated receptor alpha |
| TC11002069.hg.1 | **-0,029934** | **-0,015048** | RAB6A | RAB6A, member RAS oncogene family |
| TC01003233.hg.1 | **-0,059936** | **-0,014925** | MRPL9 | mitochondrial ribosomal protein L9 |
| TC06002270.hg.1 | **-0,033315** | **-0,007054** | TAGAP | T-cell activation RhoGTPaseactivatingprotein |
| TC13000120.hg.1 | **0,002615** | **0,026886** | NBEA | neurobeachin |
| TC02001826.hg.1 | **0,048593** | **0,0523** | FBXO11 | F-box protein 11 |
| TC02000414.hg.1 | **0,068194** | **0,078869** | PCBP1 | poly(rC) binding protein 1 |
| TC20000939.hg.1 | **0,071786** | **0,130133** | ADNP | activity-dependentneuroprotectorhomeobox |
| TC20000006.hg.1 | **0,062053** | **0,138536** | ZCCHC3 | zinc finger, CCHC domaincontaining 3 |
| TC11000954.hg.1 | **0,055422** | **0,148539** | YAP1 | Yes-associatedprotein 1 |

| Probe Set ID | **mir15 vs scramble log-ratio** | **mir15 vs scramble log-ratio** | Gene Symbol | Gene Description |
| --- | --- | --- | --- | --- |
| TC11003457.hg.1 | **-1,104408** | **-1,205423** | **ARL2** | **ADP-ribosylation factor-like 2** |
| TC01001685.hg.1 | **-0,028212** | **-0,888098** | **BTG2** | **BTG family, member 2** |
| TC10000808.hg.1 | **-0,268** | **-0,843751** | **PDCD4** | **programmed cell death 4 (neoplastic transformation inhibitor)** |
| TC19000416.hg.1 | **-0,343602** | **-0,814478** | CCNE1 | cyclin E1 |
| TC17000349.hg.1 | **-0,234493** | **-0,681441** | CPD | carboxypeptidase D |
| TC03001374.hg.1 | **-0,311853** | **-0,621196** | CDC25A | cell division cycle 25A |
| TC18000132.hg.1 | **-0,340869** | **-0,503195** | RNF125 | ring fingerprotein 125, E3 ubiquitinprotein ligase |
| TC01000302.hg.1 | **-0,386172** | **-0,475222** | LYPLA2 | lysophospholipase II |
| TC04000689.hg.1 | **-0,226494** | **-0,468351** | SCOC | short coiled-coilprotein |
| TC17001803.hg.1 | **-0,336033** | **-0,433919** | SMURF2 | SMAD specific E3 ubiquitin protein ligase 2 |
| TC18000133.hg.1 | **-0,195747** | **-0,403502** | RNF138 | ring fingerprotein 138, E3 ubiquitinprotein ligase |
| TC10000738.hg.1 | **-0,336565** | **-0,384645** | BTRC | beta-transducinrepeatcontaining E3 ubiquitinprotein ligase |
| TC12000060.hg.1 | **-0,091148** | **-0,365949** | CCND2 | cyclin D2 |
| TC16001087.hg.1 | **-0,061673** | **-0,357187** | DNAJA2 | DnaJ (Hsp40) homolog, subfamily A, member 2 |
| TC01001583.hg.1 | **-0,069154** | **-0,325196** | LAMC1 | laminin, gamma 1 (formerly LAMB2) |
| TC15000638.hg.1 | **-0,111231** | **-0,320487** | KIF23 | kinesinfamilymember 23 |
| TC14001563.hg.1 | **-0,090015** | **-0,310983** | CDCA4 | cell division cycle associated 4 |
| TC12001273.hg.1 | **-0,085459** | **-0,307993** | WBP11 | WW domain binding protein 11 |
| TC12000449.hg.1 | **-0,042985** | **-0,303615** | HOXC11 | homeobox C11 |
| TC19000747.hg.1 | **-0,13978** | **-0,294725** | AP2A1 | adaptor-relatedproteincomplex 2, alpha 1 subunit |
| TC21000039.hg.1 | **-0,178568** | **-0,266168** | USP25 | ubiquitinspecific peptidase 25 |
| TC13000663.hg.1 | **-0,239566** | **-0,250477** | KPNA3 | karyopherin alpha 3 (importin alpha 4) |
| TC17000060.hg.1 | **-0,39754** | **-0,248311** | KIF1C | kinesinfamilymember 1C |
| TC11003470.hg.1 | **-0,159008** | **-0,247922** | CHEK1 | checkpoint kinase 1 |
| TC01000125.hg.1 | **-0,280384** | **-0,220205** | UBE4B | ubiquitination factor E4B |
| TC17001874.hg.1 | **-0,003892** | **-0,21951** | GGA3 | golgi-associated, gamma adaptin ear containing, ARF binding protein 3 |
| TC20001751.hg.1 | **-0,224512** | **-0,212294** | UBE2V1 | ubiquitin-conjugating enzyme E2 variant 1 |
| TC20000056.hg.1 | **-0,040679** | **-0,211092** | CDS2 | CDP-diacylglycerol synthase (phosphatidatecytidylyltransferase) 2 |
| TC01000358.hg.1 | **-0,186137** | **-0,210508** | WDTC1 | WD and tetratricopeptiderepeats 1 |
| TC01001724.hg.1 | **-0,34287** | **-0,208828** | RASSF5 | Ras association (RalGDS/AF-6) domainfamilymember 5 |
| TC02002521.hg.1 | **-0,171369** | **-0,205335** | TLK1 | tousled-like kinase 1 |
| TC12001391.hg.1 | **-0,27829** | **-0,198468** | KIF21A | kinesinfamilymember 21A |
| TC17000538.hg.1 | **-0,182479** | **-0,191822** | PSME3 | proteasome (prosome, macropain) activator subunit 3 (PA28 gamma; Ki) |
| TC18000003.hg.1 | **-0,188248** | **-0,191381** | USP14 | ubiquitinspecific peptidase 14 (tRNA-guanine transglycosylase) |
| TC15000448.hg.1 | **-0,062544** | **-0,184296** | RNF111 | ring fingerprotein 111 |
| TC17001961.hg.1 | **-0,117407** | **-0,184008** | ARHGDIA | Rho GDP dissociation inhibitor (GDI) alpha |
| TC10001173.hg.1 | **-0,287005** | **-0,18017** | CUL2 | cullin 2 |
| TC02002111.hg.1 | **-0,002511** | **-0,177398** | LMAN2L | lectin, mannose-binding 2-like |
| TC16000550.hg.1 | **-0,183155** | **-0,171813** | PSKH1 | protein serine kinase H1 |
| TC17000619.hg.1 | **-0,012741** | **-0,170091** | NFE2L1 | nuclear factor (erythroid-derived 2)-like 1 |
| TC02000439.hg.1 | **-0,044337** | **-0,16977** | SMYD5 | SMYD familymember 5 |
| TC01002864.hg.1 | **-0,028256** | **-0,162821** | TGFBR3 | transforming growth factor, beta receptor III |
| TC15001342.hg.1 | **-0,100584** | **-0,154172** | COPS2 | COP9 signalosomesubunit 2 |
| TC15000613.hg.1 | **-0,027095** | **-0,150552** | MAP2K1 | mitogen-activated protein kinase kinase 1 |
| TC03001372.hg.1 | **-0,155951** | **-0,148143** | MAP4 | microtubule-associatedprotein 4 |
| TC12000089.hg.1 | **-0,120742** | **-0,144002** | COPS7A | COP9 signalosomesubunit 7A |
| TC14000916.hg.1 | **-0,000843** | **-0,142836** | SUPT16H | suppressor of Ty 16 homolog (S. cerevisiae) |
| TC10001526.hg.1 | **-0,035048** | **-0,141542** | CPEB3 | cytoplasmicpolyadenylationelement binding protein 3 |
| TC11000656.hg.1 | **-0,031404** | **-0,140696** | KLC2 | kinesin light chain 2 |
| TC12001598.hg.1 | **-0,259489** | **-0,139032** | RNF41 | ring fingerprotein 41 |
| TC10000609.hg.1 | **-0,143243** | **-0,135505** | BMPR1A | bone morphogenetic protein receptor, type IA |
| TC04000396.hg.1 | **-0,150009** | **-0,133096** | SLC4A4 | solute carrier family 4, sodium bicarbonate cotransporter, member 4 |
| TC02000512.hg.1 | **-0,028242** | **-0,132699** | VAMP8 | vesicle-associated membrane protein 8 |
| TC20000216.hg.1 | **-0,03863** | **-0,13223** | MAPRE1 | microtubule-associated protein, RP/EB family, member 1 |
| TC01003778.hg.1 | **-0,139237** | **-0,128988** | PLXNA2 | plexin A2 |
| TC11002062.hg.1 | **-0,30908** | **-0,122628** | FCHSD2 | FCH and double SH3 domains 2 |
| TC01000947.hg.1 | **-0,060719** | **-0,120425** | GNAI3 | guanine nucleotide binding protein (G protein), alpha inhibiting activity polypeptide 3 |
| TC02001560.hg.1 | **-0,05958** | **-0,118302** | YWHAQ | tyrosine 3-monooxygenase/tryptophan 5-monooxygenase activation protein, theta polypeptide |
| TC11001002.hg.1 | **-0,131871** | **-0,114616** | DIXDC1 | DIX domaincontaining 1 |
| TC19000118.hg.1 | **-0,069311** | **-0,114225** | TRIP10 | thyroid hormone receptorinteractor 10 |
| TC14001009.hg.1 | **-0,160387** | **-0,103238** | HECTD1 | HECT domaincontaining E3 ubiquitinprotein ligase 1 |
| TC02001395.hg.1 | **-0,039501** | **-0,097727** | COPS7B | COP9 signalosomesubunit 7B |
| TC11002185.hg.1 | **-0,079369** | **-0,095689** | CHORDC1 | cysteine and histidine-rich domain (CHORD) containing 1 |
| TC04000257.hg.1 | **-0,017327** | **-0,087014** | TMEM33 | transmembraneprotein 33 |
| TC14000238.hg.1 | **-0,226023** | **-0,082808** | MIPOL1 | mirror-image polydactyly 1 |
| TC22001489.hg.1 | **-0,130286** | **-0,081782** | PISD | phosphatidylserinedecarboxylase |
| TC21000346.hg.1 | **-0,167511** | **-0,081151** | ADAMTS5 | ADAM metallopeptidase with thrombospondin type 1 motif, 5 |
| TC03000182.hg.1 | **-0,051211** | **-0,07277** | CTDSPL | CTD (carboxy-terminal domain, RNA polymerase II, polypeptide A) small phosphatase-like |
| TC01000280.hg.1 | **-0,0573** | **-0,070665** | CDC42 | cell division cycle 42 |
| TC10001089.hg.1 | **-0,060862** | **-0,070645** | NEBL | nebulette |
| TC02001199.hg.1 | **-0,124132** | **-0,069369** | CD28 | CD28 molecule |
| TC03000627.hg.1 | **-0,048036** | **-0,068607** | CASR | calcium-sensingreceptor |
| TC01001883.hg.1 | **-0,182571** | **-0,066049** | WNT3A | wingless-type MMTV integration site family, member 3A |
| TC12000555.hg.1 | **-0,108205** | **-0,065578** | USP15 | ubiquitinspecific peptidase 15 |
| TC01000127.hg.1 | **-0,09673** | **-0,063052** | KIF1B | kinesinfamilymember 1B |
| TC17001493.hg.1 | **-0,035971** | **-0,061213** | KRTAP4-4 | keratinassociatedprotein 4-4 |
| TC16000488.hg.1 | **-0,211679** | **-0,057358** | CX3CL1 | chemokine (C-X3-C motif) ligand 1 |
| TC17001734.hg.1 | **-0,027537** | **-0,056249** | MTMR4 | myotubularinrelatedprotein 4 |
| TC17000994.hg.1 | **-0,068002** | **-0,055289** | MNT | MNT, MAX dimerizationprotein |
| TC12001168.hg.1 | **-0,097519** | **-0,054077** | SLC2A14 | solute carrier family 2 (facilitated glucose transporter), member 14 |
| TC16001288.hg.1 | **-0,083522** | **-0,053361** | ADAMTS18 | ADAM metallopeptidase with thrombospondin type 1 motif, 18 |
| TC17000402.hg.1 | **-0,099619** | **-0,049059** | TAF15 | TAF15 RNA polymerase II, TATA box binding protein (TBP)-associated factor, 68kDa |
| TC11001975.hg.1 | **-0,011237** | **-0,046962** | SPTBN2 | spectrin, beta, non-erythrocytic 2 |
| TC01000800.hg.1 | **-0,048032** | **-0,043051** | LPHN2 | latrophilin 2 |
| TC12001341.hg.1 | **-0,084533** | **-0,041906** | PTHLH | parathyroid hormone-like hormone |
| TC03001604.hg.1 | **-0,028014** | **-0,039015** | DCBLD2 | discoidin, CUB and LCCL domain containing 2 |
| TC14000362.hg.1 | **-0,02072** | **-0,035437** | PPM1A | protein phosphatase, Mg2+/Mn2+ dependent, 1A |
| TC15000299.hg.1 | **-0,097003** | **-0,026681** | DLL4 | delta-like 4 (Drosophila) |
| TC13000717.hg.1 | **-0,117295** | **-0,0265** | PCDH9 | protocadherin 9 |
| TC17000024.hg.1 | **-0,209062** | **-0,019509** | PAFAH1B1 | platelet-activating factor acetylhydrolase 1b, regulatory subunit 1 (45kDa) |
| TC14000773.hg.1 | **-0,06049** | **-0,019414** | PPP2R5C | protein phosphatase 2, regulatory subunit B', gamma |
| TC01002328.hg.1 | **-0,080089** | **-0,018463** | HSPG2 | heparan sulfate proteoglycan 2 |
| TC03000579.hg.1 | **-0,080475** | **-0,015355** | SIDT1 | SID1 transmembranefamily, member 1 |
| TC19002634.hg.1 | **-0,116375** | **-0,014628** | EGLN2 | egl nine homolog 2 (C. elegans) |
| TC03000024.hg.1 | **-0,064074** | **-0,014622** | GRM7 | glutamate receptor, metabotropic 7 |
| TC16001102.hg.1 | **-0,203964** | **-0,013044** | ZNF423 | zinc fingerprotein 423 |
| TC01001591.hg.1 | **-0,127055** | **-0,012648** | C1orf21 | chromosome 1 open reading frame 21 |
| TC12000534.hg.1 | **-0,205722** | **-0,010745** | KIF5A | kinesinfamilymember 5A |
| TC03001160.hg.1 | **-0,123404** | **-0,009798** | ATP2B2 | ATPase, Ca++ transporting, plasma membrane 2 |
| TC01000998.hg.1 | **-0,107297** | **-0,007621** | LRIG2 | leucine-rich repeats and immunoglobulin-like domains 2 |
| TC02001017.hg.1 | **-0,220355** | **-0,006955** | GORASP2 | golgi reassembly stacking protein 2, 55kDa |
| TC12001607.hg.1 | **-0,163005** | **-0,004412** | GLS2 | glutaminase 2 (liver, mitochondrial) |
| TC12001972.hg.1 | **-0,241311** | **-0,003366** | ATXN2 | ataxin 2 |
| TC16001347.hg.1 | **-0,156453** | **-0,000837** | CBFA2T3 | core-binding factor, runt domain, alpha subunit 2; translocated to, 3 |
| TC16000154.hg.1 | **-0,269787** | **-0,000458** | ATF7IP2 | activating transcription factor 7 interacting protein 2 |
| TC03001532.hg.1 | **0,055091** | **0,000433** | LRIG1 | leucine-rich repeats and immunoglobulin-like domains 1 |
| TC17001890.hg.1 | **0,070703** | **0,004459** | ACOX1 | acyl-CoAoxidase 1, palmitoyl |
| TC16000176.hg.1 | **0,244521** | **0,004651** | BFAR | bifunctionalapoptosisregulator |
| TC01003005.hg.1 | **0,613554** | **0,009894** | RSBN1 | round spermatid basic protein 1 |
| TC20000900.hg.1 | **0,159326** | **0,01416** | ELMO2 | engulfment and cellmotility 2 |
| TC19000792.hg.1 | **0,128593** | **0,022227** | PPP2R1A | protein phosphatase 2, regulatory subunit A, alpha |
| TC01003417.hg.1 | **0,088668** | **0,022292** | DEDD | deatheffectordomaincontaining |
| TC15000386.hg.1 | **0,045283** | **0,027736** | FGF7 | fibroblastgrowth factor 7 |
| TC10000886.hg.1 | **0,177203** | **0,028761** | PLEKHA1 | pleckstrin homology domain containing, family A (phosphoinositide binding specific) member 1 |
| TC17001552.hg.1 | **0,129105** | **0,029079** | VAT1 | vesicle amine transport protein 1 homolog (T. californica) |
| TC03000229.hg.1 | **0,400794** | **0,031401** | SNRK | SNF related kinase |
| TC19000727.hg.1 | **0,002008** | **0,032052** | PPFIA3 | protein tyrosine phosphatase, receptor type, f polypeptide (PTPRF), interactingprotein (liprin), alpha 3 |
| TC02001206.hg.1 | **0,043737** | **0,033481** | NRP2 | neuropilin 2 |
| TC11001386.hg.1 | **0,015527** | **0,044017** | STK33 | serine/threonine kinase 33 |
| TC21000130.hg.1 | **0,322722** | **0,045753** | SON | SON DNA binding protein |
| TC20000006.hg.1 | **0,148435** | **0,052579** | ZCCHC3 | zinc finger, CCHC domaincontaining 3 |
| TC12001138.hg.1 | **0,31818** | **0,05498** | VAMP1 | vesicle-associated membrane protein 1 (synaptobrevin 1) |
| TC03000744.hg.1 | **0,224876** | **0,055332** | MRAS | muscle RAS oncogenehomolog |
| TC03001956.hg.1 | **0,147802** | **0,056354** | KPNA4 | karyopherin alpha 4 (importin alpha 3) |
| TC02000230.hg.1 | **0,050392** | **0,059379** | CRIM1 | cysteine rich transmembrane BMP regulator 1 (chordin-like) |
| TC22000470.hg.1 | **0,027807** | **0,060931** | CECR6 | cat eye syndrome chromosome region, candidate 6 |
| TC11002275.hg.1 | **0,093892** | **0,068411** | ARHGAP20 | Rho GTPaseactivatingprotein 20 |
| TC11001435.hg.1 | **0,180524** | **0,073596** | PTH | parathyroid hormone |
| TC17000813.hg.1 | **0,050439** | **0,076313** | KCNJ2 | potassium inwardly-rectifying channel, subfamily J, member 2 |
| TC10001486.hg.1 | **0,517217** | **0,077151** | GLUD1 | glutamate dehydrogenase 1 |
| TC01002948.hg.1 | **0,037405** | **0,096638** | WDR47 | WD repeatdomain 47 |
| TC13000860.hg.1 | **0,182269** | **0,0979** | EFNB2 | ephrin-B2 |
| TC15001558.hg.1 | **0,021467** | **0,102299** | RASL12 | RAS-like, family 12 |
| TC14001539.hg.1 | **0,34028** | **0,126756** | BAG5 | BCL2-associated athanogene 5 |
| TC15000622.hg.1 | **0,027376** | **0,127131** | SMAD3 | SMAD familymember 3 |
| TC17001340.hg.1 | **1,110095** | **0,146148** | OMG | oligodendrocyte myelinglycoprotein |
| TC16000576.hg.1 | **0,158853** | **0,148289** | PDPR | pyruvate dehydrogenase phosphatase regulatory subunit |
| TC16001095.hg.1 | **0,083457** | **0,165763** | SIAH1 | siah E3 ubiquitinprotein ligase 1 |
| TC20000905.hg.1 | **0,117625** | **0,210077** | SLC13A3 | solute carrier family 13 (sodium-dependentdicarboxylate transporter), member 3 |
| TC12001251.hg.1 | **0,104378** | **0,289476** | LRP6 | low density lipoprotein receptor-related protein 6 |

**Supplemental Table S4. List of downregulated mRNAs after *miR-15a-5p* or *miR-21-5p* overexpression in K562 cells.**

**
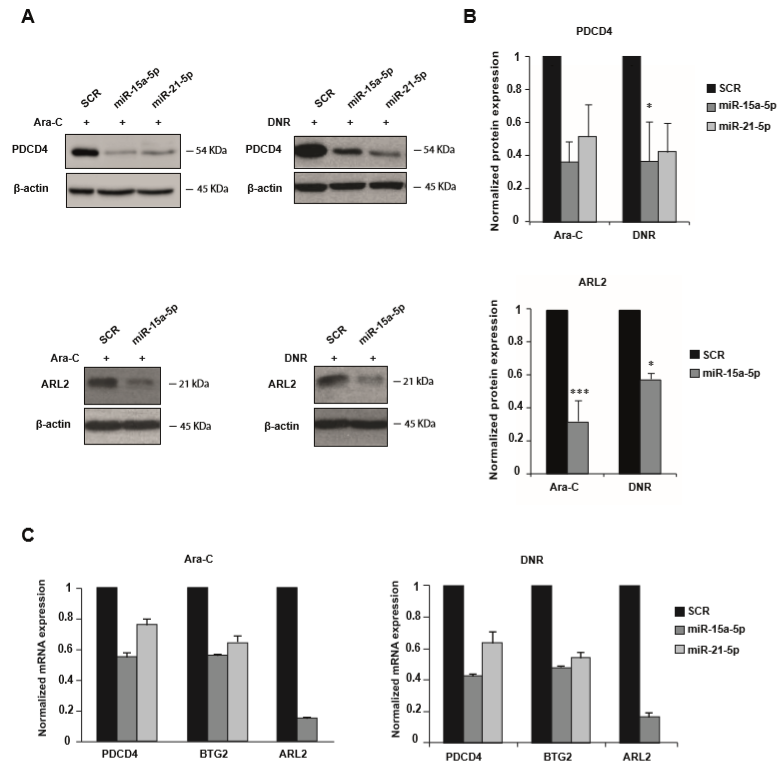
**

**Supplemental Figure S9. *miR-15a-5p* and *miR-21-5p* regulate the expression of *ARL2*, *PDCD4* and *BTG2***. (**A**) Western blotting of PDCD4 and ARL2 protein expression in K562 cells treated with cytarabine or daunorubicin after 24 hours of transfection with synthetic miR-15a-5p, miR-21-5p or SCR. The protein loading control was performed using β-actin. (**B**) Bands were quantified by densitometry using Image J software. Data represent the average of 3 independent experiments ± SD. *P* values were obtained using a *t* test. * Indicates a significant difference *p ≤ 0.05* and *** indicates a significant difference *p ≤ 0.001*. (**C**) Quantitative RT-PCR of *PDCD4*, *ARL2* and *BTG2* after 24 hours of transfection with synthetic miR-15a-5p, miR-21-5p or SCR treated with cytarabine or daunorubicin. The results are shown as average mRNA expression after normalization with *GAPDH* and 2ΔC_t_ calculations. One representative experiment is shown.


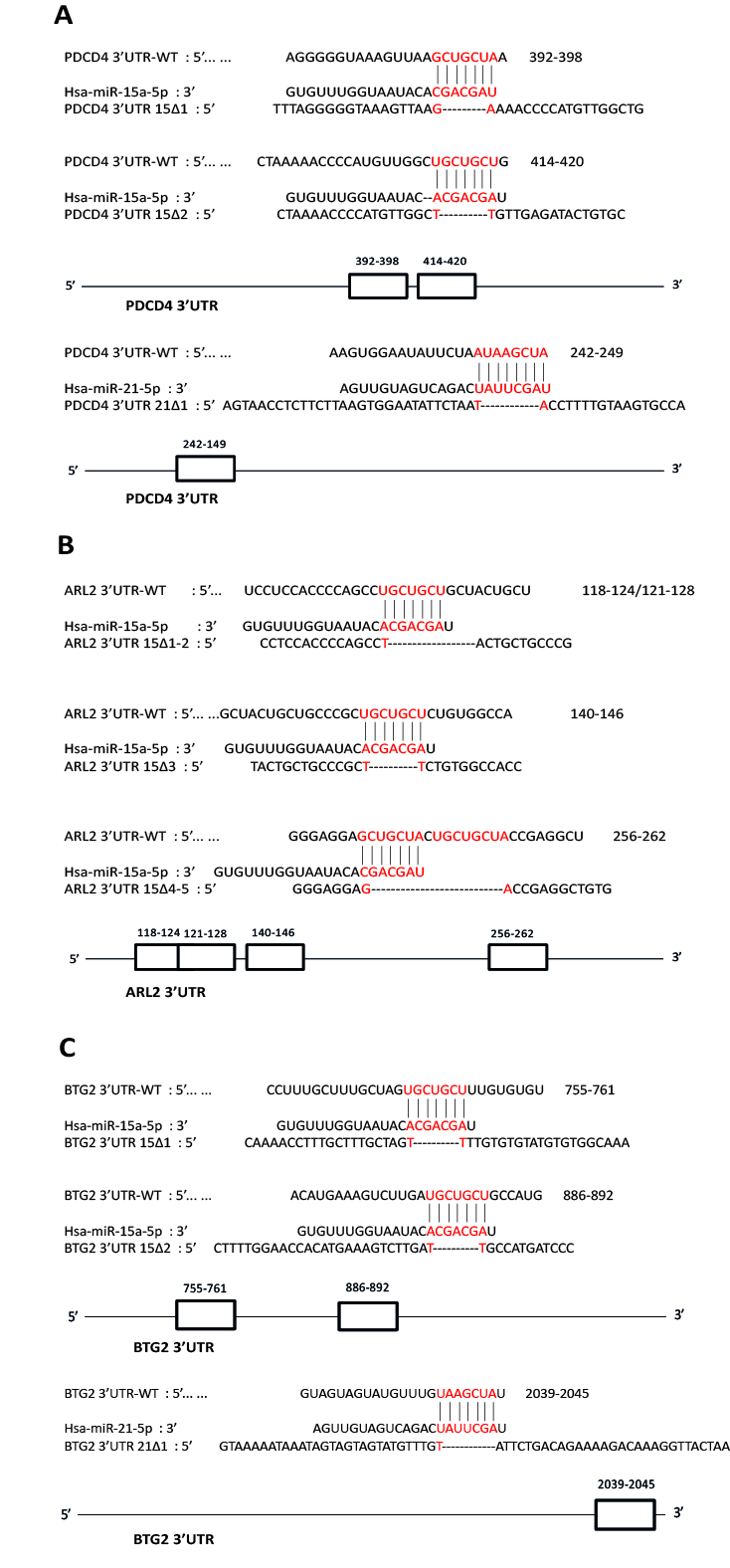


**Supplemental Figure S10.** **miRNA seed sequences in the 3’ UTRs of target genes.** Predicted binding sites of *miR-15a-5p* in the *PDCD4* (**A**), *BTG2* (**B**) and *ARL2* (**C**) 3′ UTR mRNA (red: nucleotides involved in the target interaction). Deletions of *miR-15a-5p* and *miR-21-5p* seed-matching sites in the 3’UTRs of target genes. In the figure is shown the alignment of the seed regions of miRNAs with the

respective target 3’ UTR. The sites of target mutagenesis are indicated in red. Diagram illustrating the structure of the *PDCD4* (**A**)*, BTG2* (**B**) and *ARL2* (**C**) 3′ UTR were added.


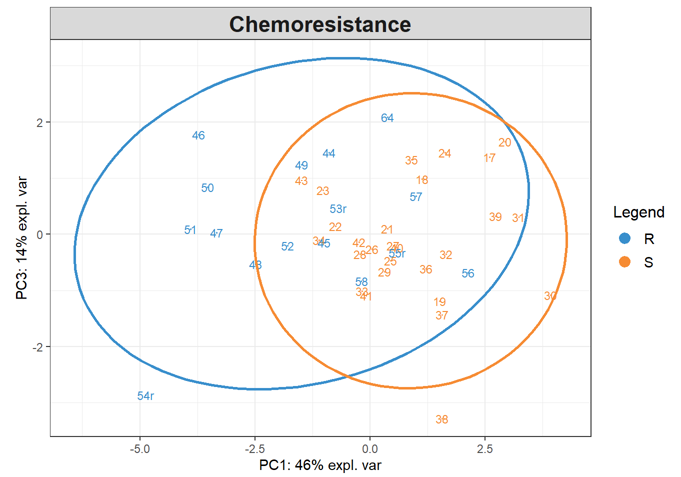


**Supplemental Figure S11.** PCA analysis based on selected gene and miRNA

expression data. The PC3 versus PC1 plot captured the separation of the same

chemoresistant patients that were clearly separated from the chemosensitive

patients in the PC2 versus PC1 plot. R for Resistant and S for Sensitive.


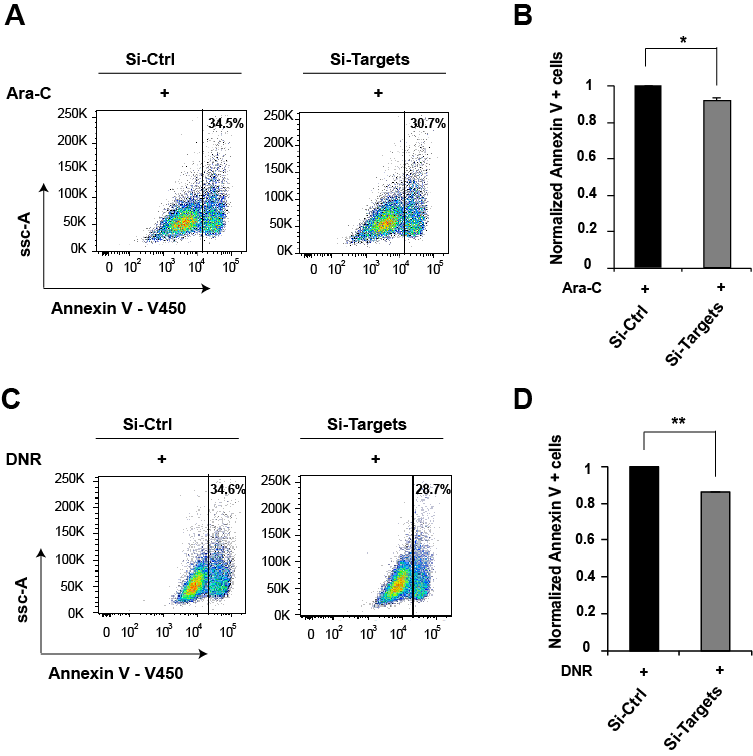


**Supplemental Figure S12. *miR-15a-5p* and *miR-21-5p* induce chemoresistance by targeting *ARL2*, *PDCD4* and *BTG2***. K562 cell lines were transfected with a combination of three siRNAs against the three target genes (siPDCD4, siARL2, siBTG2) at a concentration of 50 nM for 48 hours. K562 were treated with cytarabine (Ara-C, **A - B**) alone or daunorubicin (DNR, **C - D**) alone the last 24 hours. The results are presented as percentage of apoptotic cells. One representative experiment is showed in **(A - C)** and the average of 3 independent experiments ± SD in **(B - D)**. *P* values were obtained using *t* test. * Indicates a significant difference *p ≤ 0.05* and ** indicates a significant difference *p ≤ 0.01.*

### References

1. fastqc: http:/[www.bioinformatics.babraham.ac.uk/projects/fastqc/](file:///F:\Papier%20Blood\JCMM%20submission\Resubmission\www.bioinformatics.babraham.ac.uk\projects\fastqc\). Accessed.

2. Martin M. Cutadapt removes adapter sequences from high-throughput sequencing reads. *2011.* 2011;17(1):3.

3. Li H, Durbin R. Fast and accurate long-read alignment with Burrows-Wheeler transform. *Bioinformatics.* 2010;26(5):589-595.

4. *Rsamtools: Binary alignment (BAM), FASTA, variant call (BCF), and tabix file import. R package version 1.18.3.* [computer program]. 2019.

5. Lawrence M, Huber W, Pages H, et al. Software for computing and annotating genomic ranges. *PLoS Comput Biol.* 2013;9(8):e1003118.

6. Robinson MD, McCarthy DJ, Smyth GK. edgeR: a Bioconductor package for differential expression analysis of digital gene expression data. *Bioinformatics.* 2010;26(1):139-140.

7. *Genefilter: Methods for filtering genes from microarray experiments. R package version, 1(0)* [computer program]. 2011.

8. *Package MLInterfaces 2013* [computer program]. 2013.

9. Smyth GK. limma: Linear Models for Microarray Data. In: Gentleman R, Carey VJ, Huber W, Irizarry RA, Dudoit S, eds. *Bioinformatics and Computational Biology Solutions Using R and Bioconductor.* New York, NY: Springer New York; 2005:397-420.

10. Noel LA, Arts FA, Montano-Almendras CP, et al. The tyrosine phosphatase SHP2 is required for cell transformation by the receptor tyrosine kinase mutants FIP1L1-PDGFRalpha and PDGFRalpha D842V. *Mol Oncol.* 2014;8(3):728-740.

11. Huang da W, Sherman BT, Lempicki RA. Systematic and integrative analysis of large gene lists using DAVID bioinformatics resources. *Nat Protoc.* 2009;4(1):44-57.

12. Arts FA, Velghe AI, Stevens M, Renauld JC, Essaghir A, Demoulin JB. Idiopathic basal ganglia calcification-associated PDGFRB mutations impair the receptor signalling. *J Cell Mol Med.* 2015;19(1):239-248.
